# Supplementary figures and images for: The relationship between price and nutritional balance for young adults in the menus of Japanese restaurants
Source: PeerJ. 2024 Sep 20;12:e18091. doi: 10.7717/peerj.18091 (PMC11418817; doi:10.7717/peerj.18091)

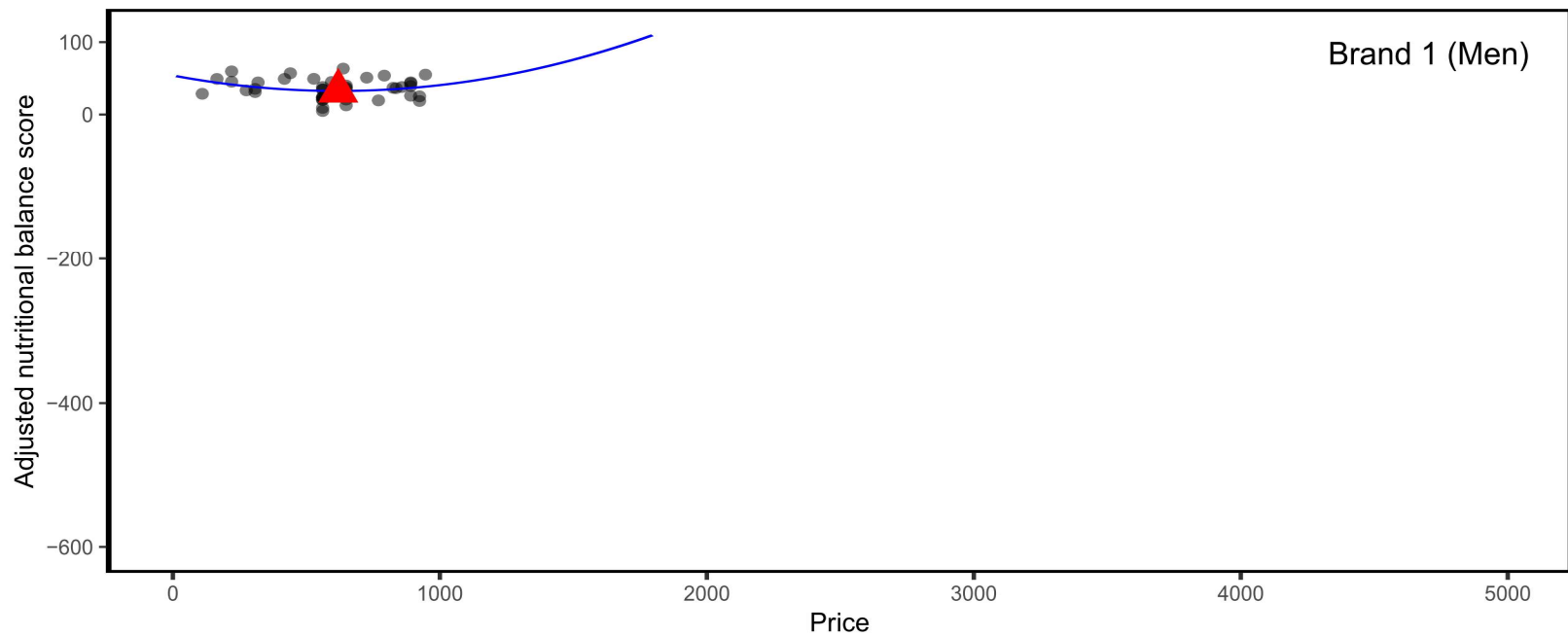

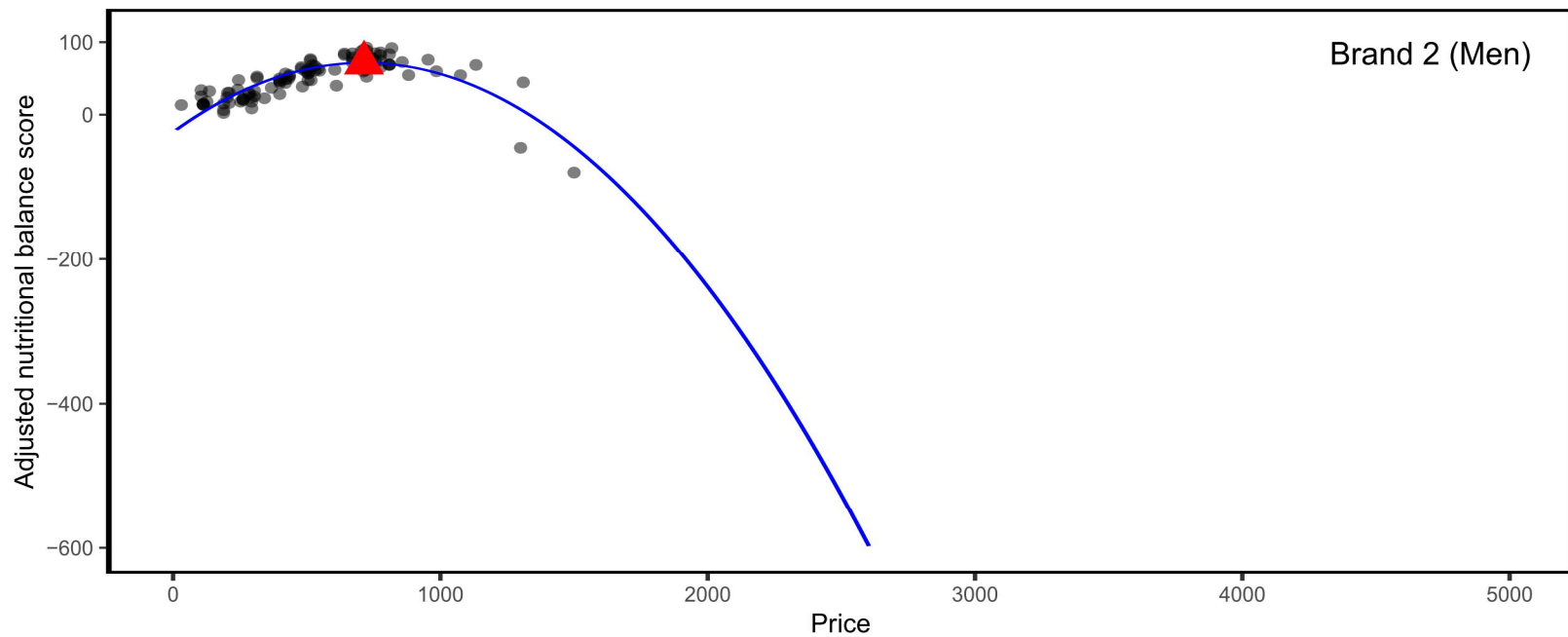

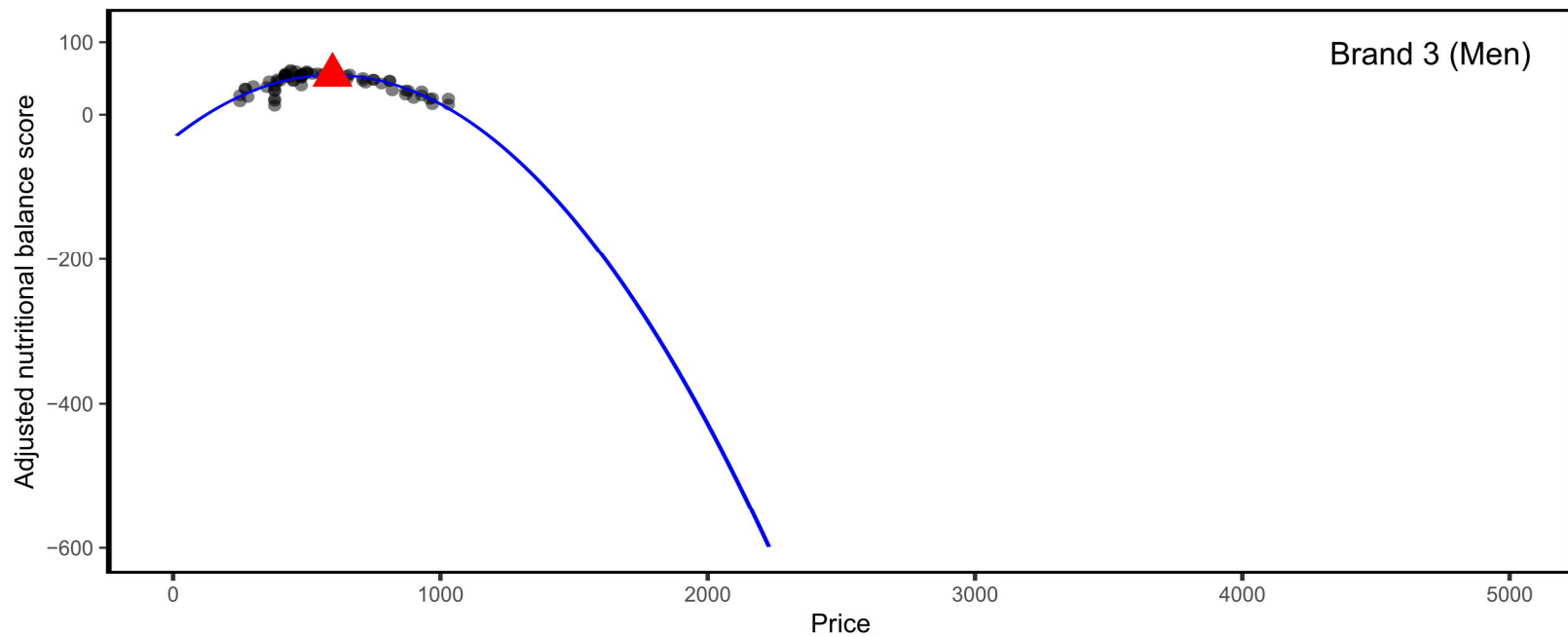

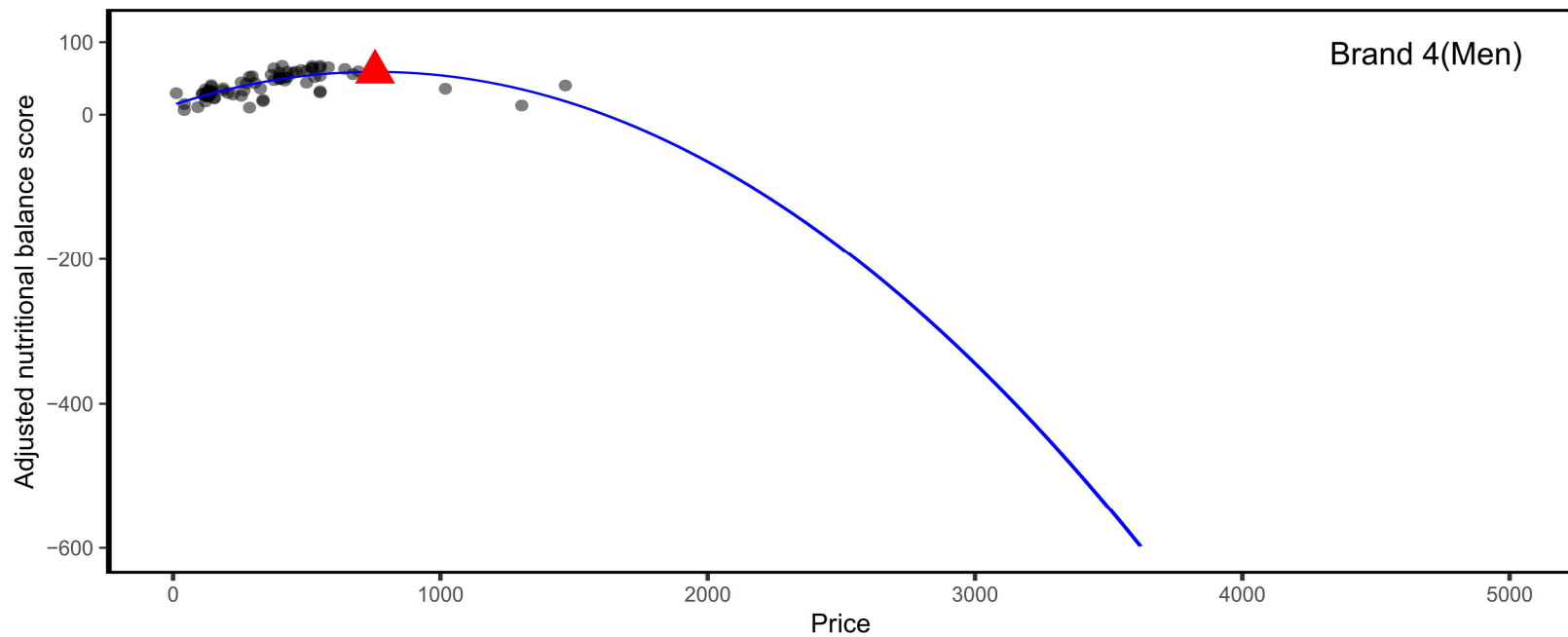

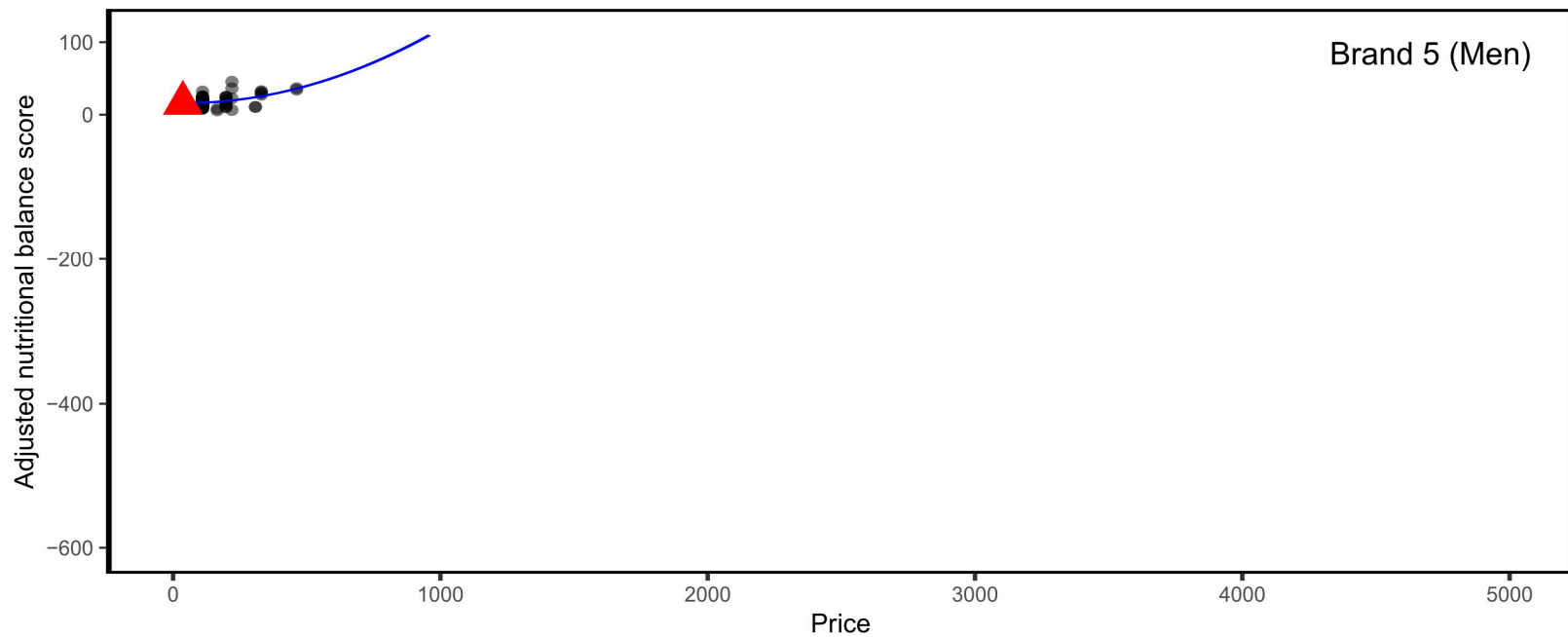

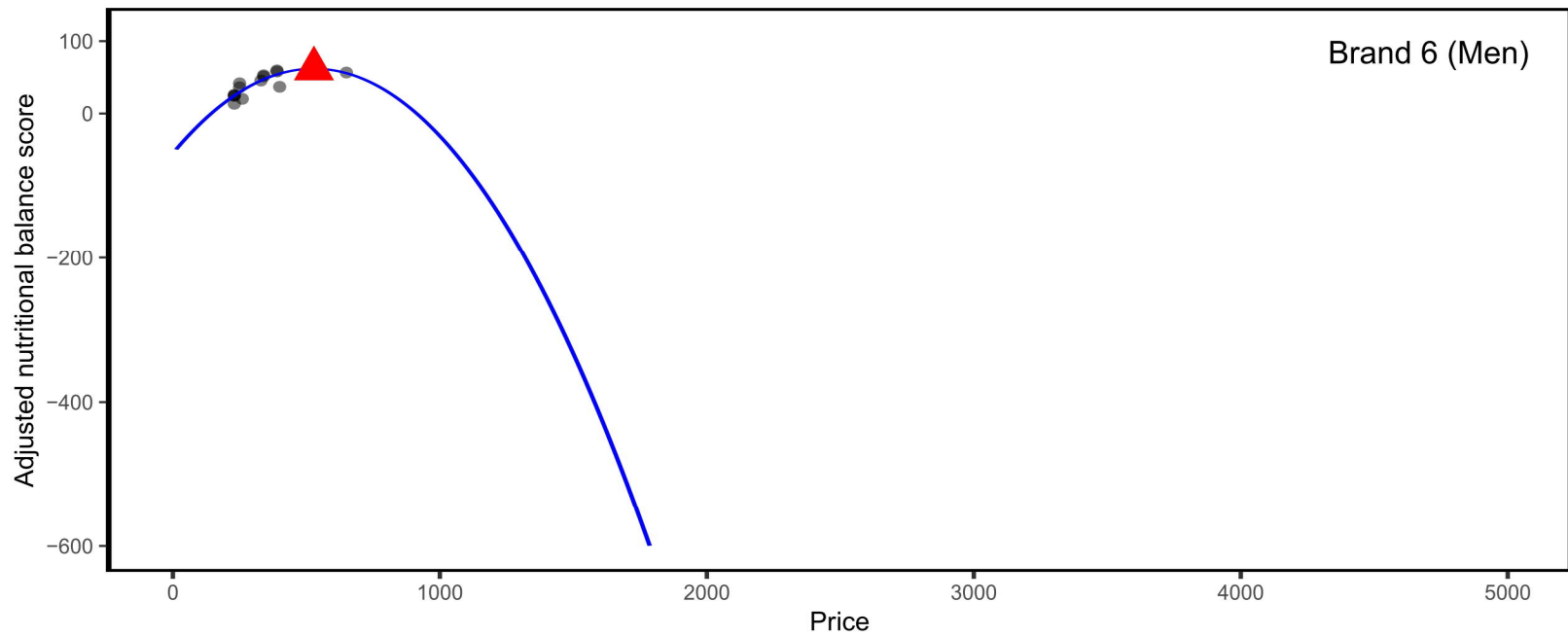

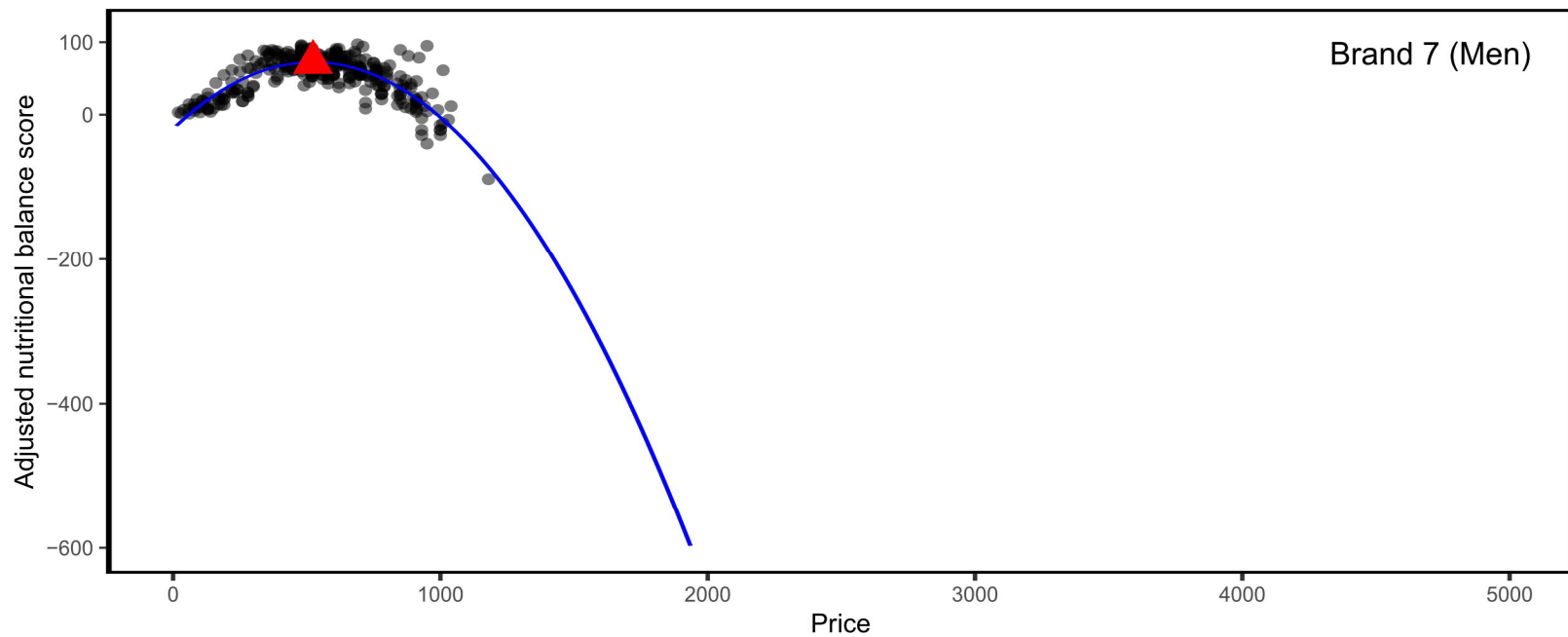

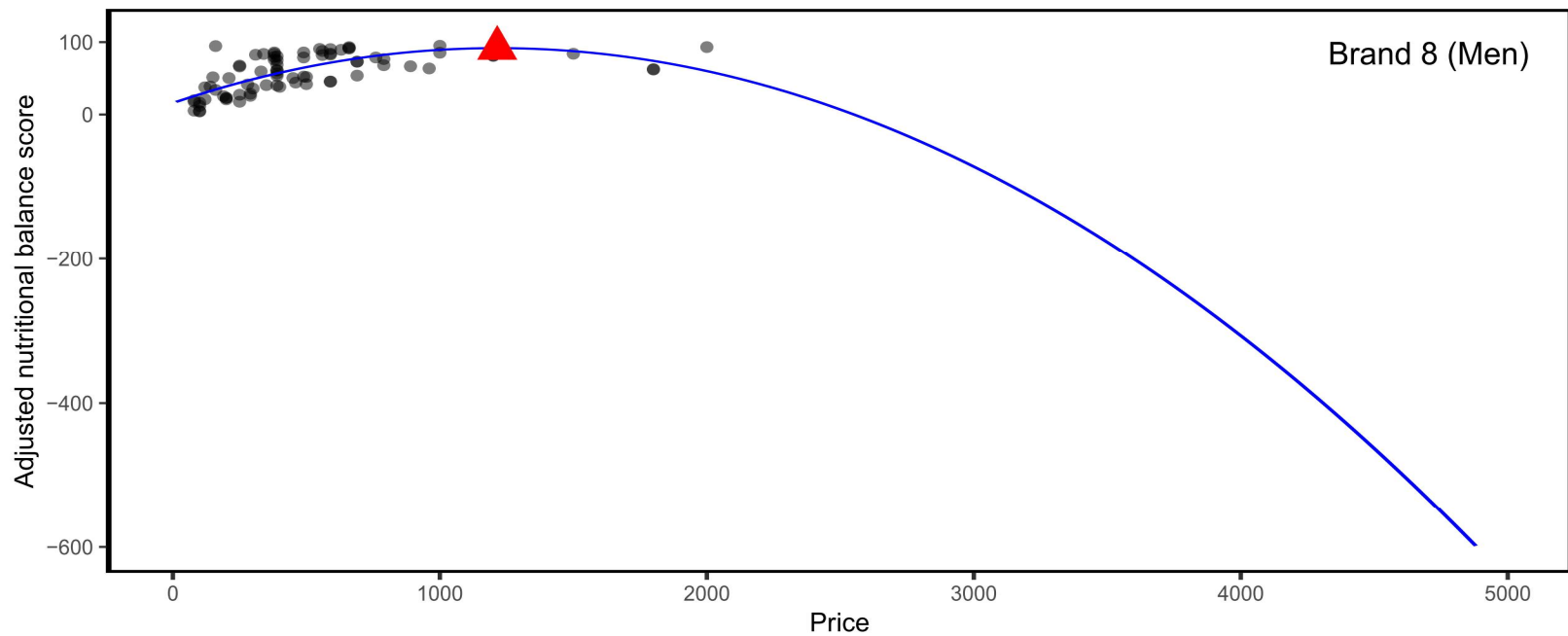

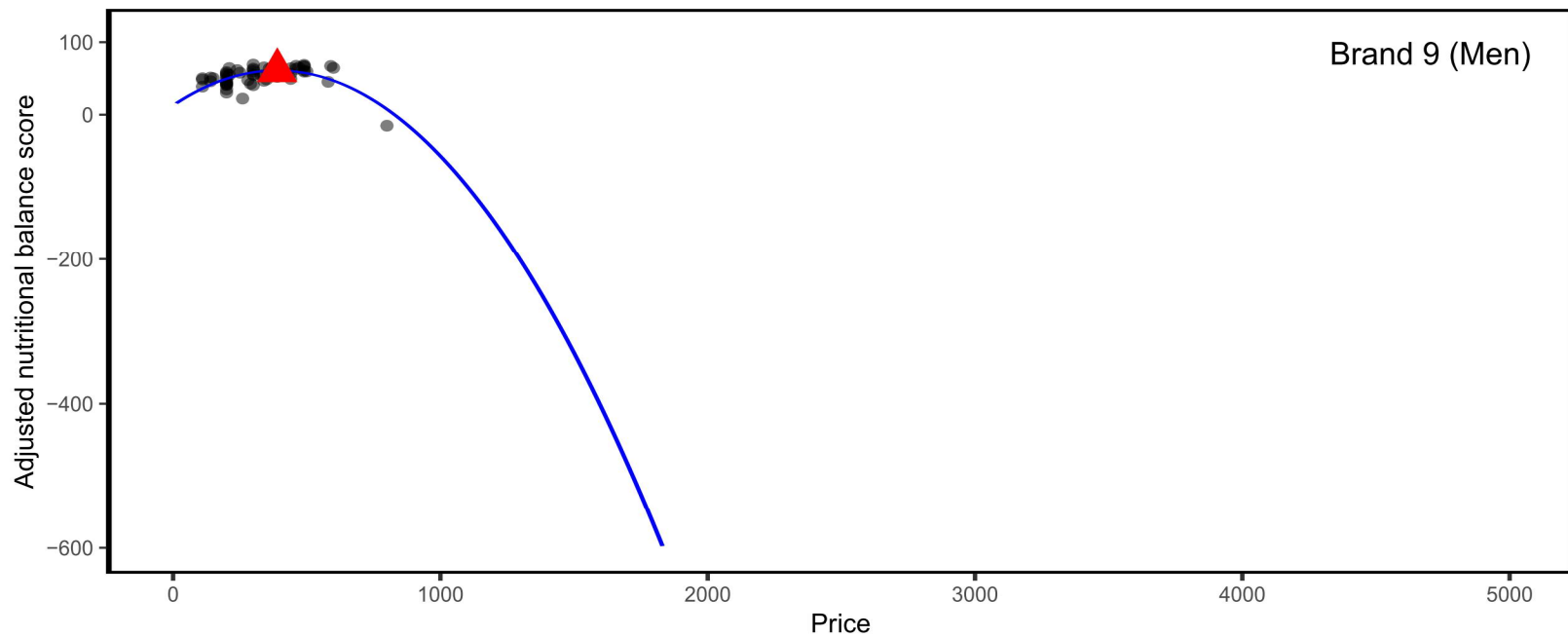

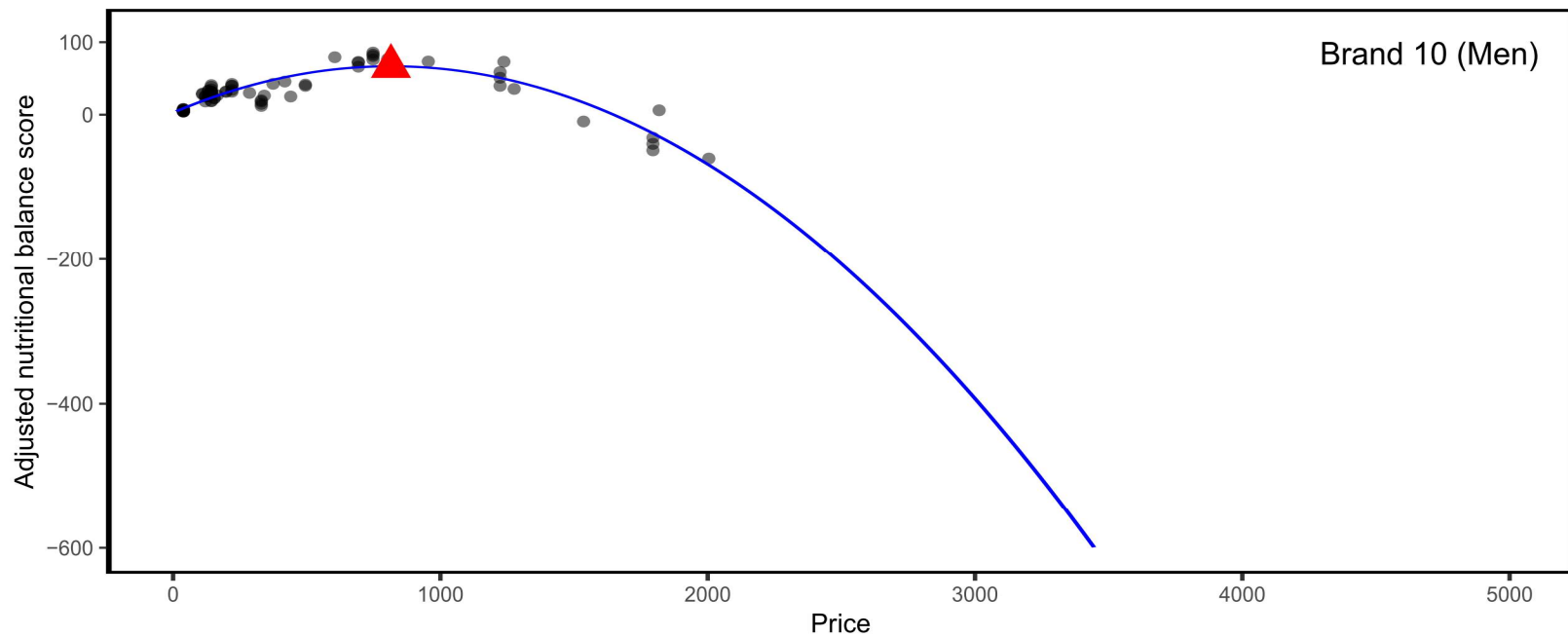

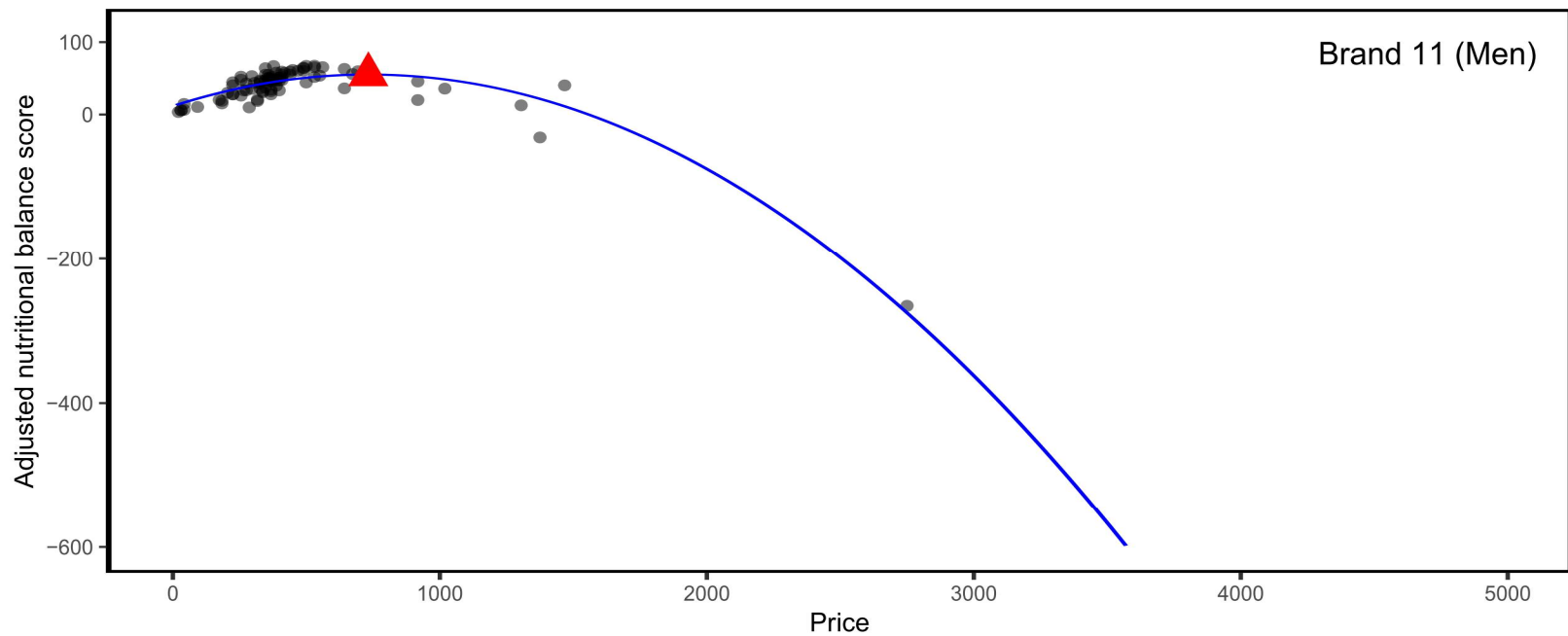

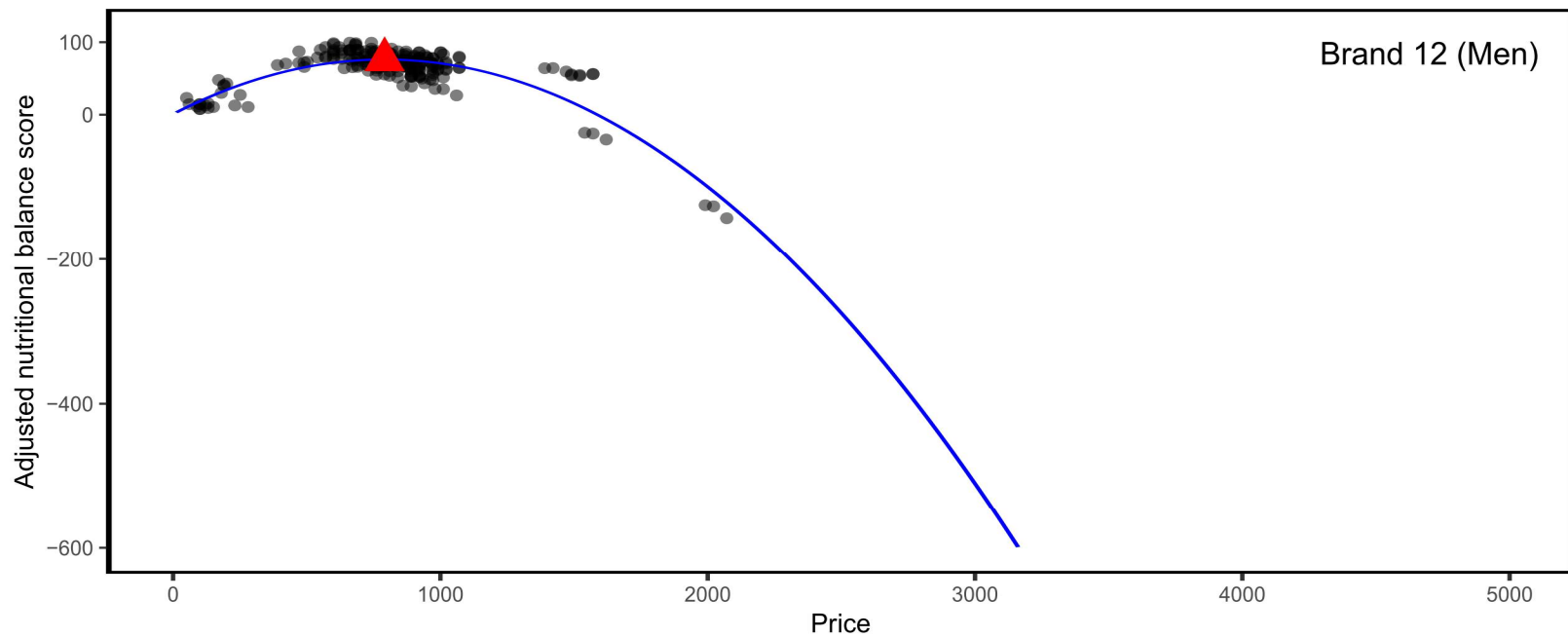

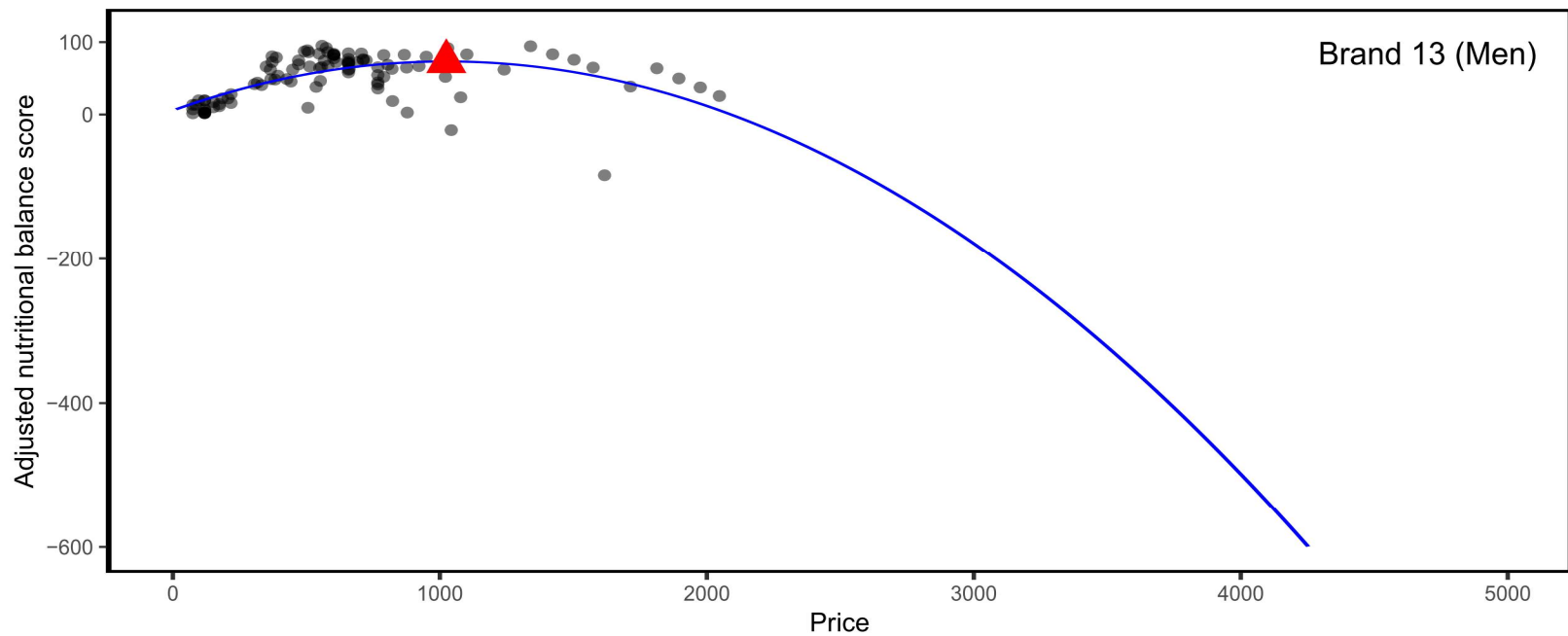

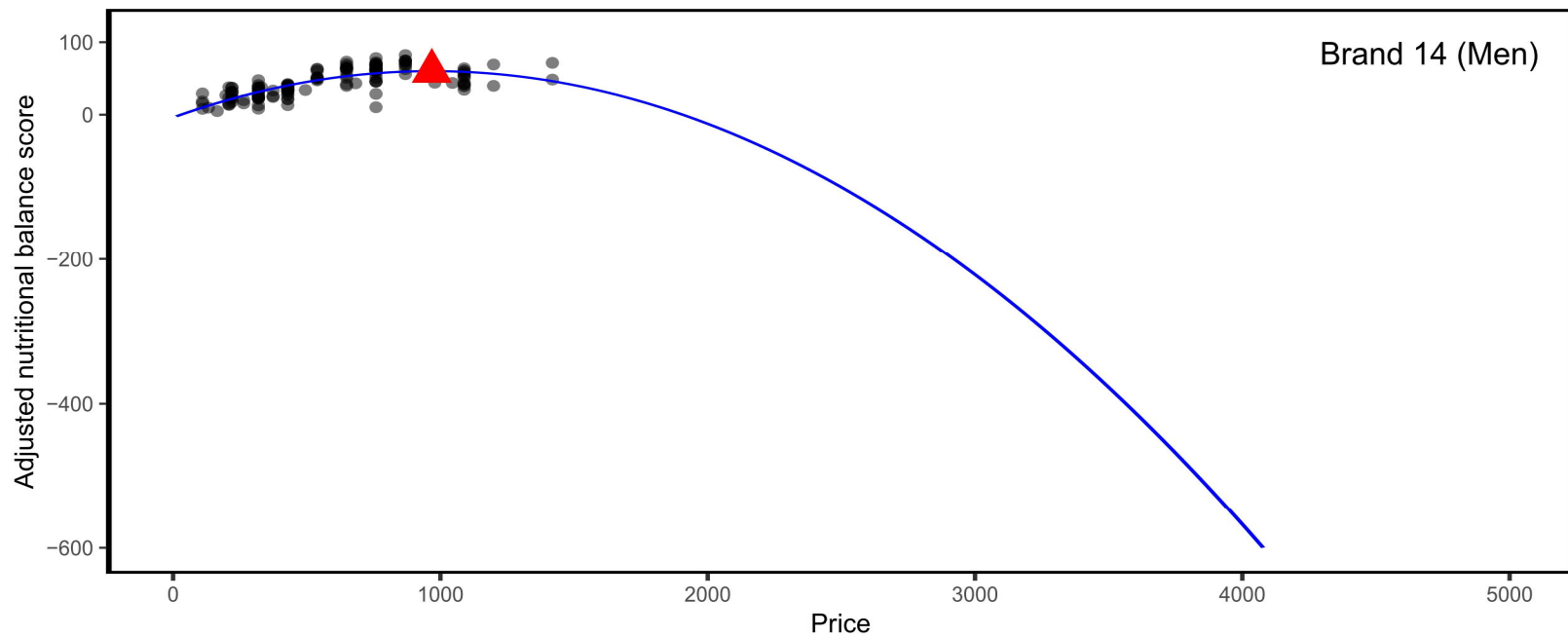

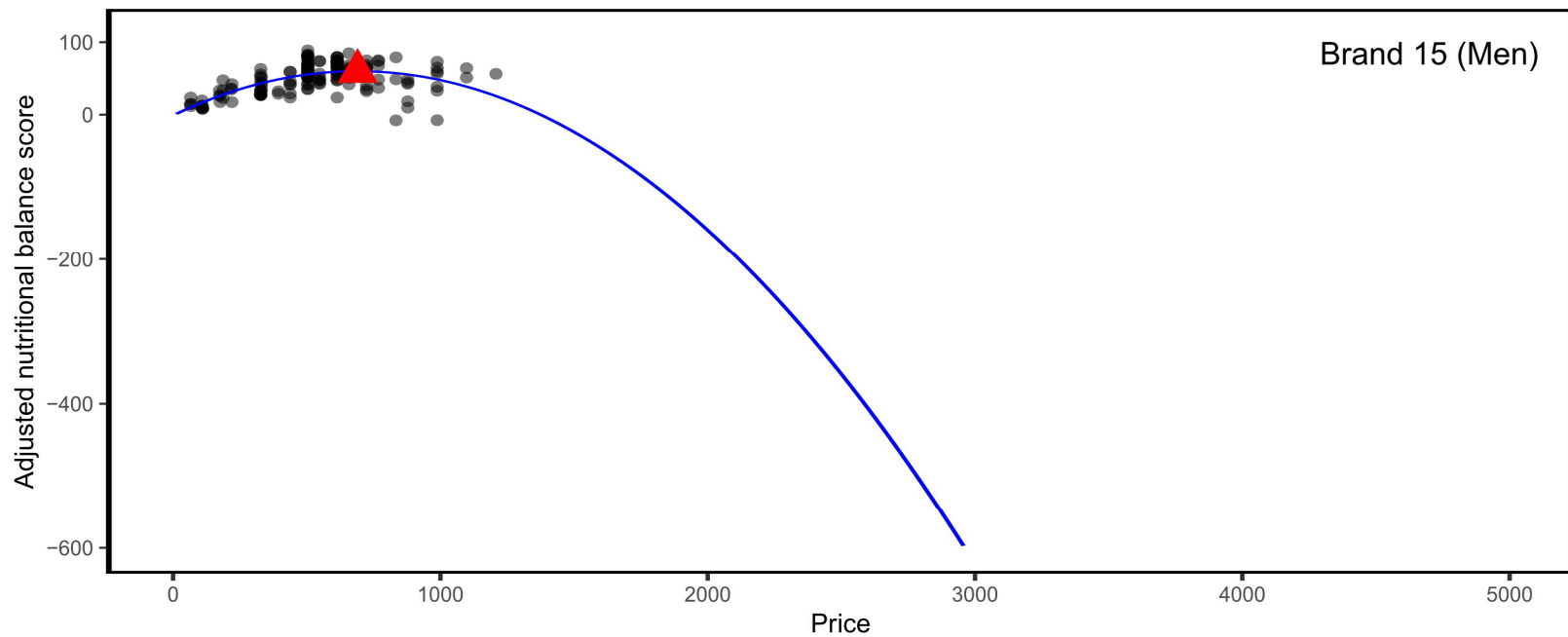

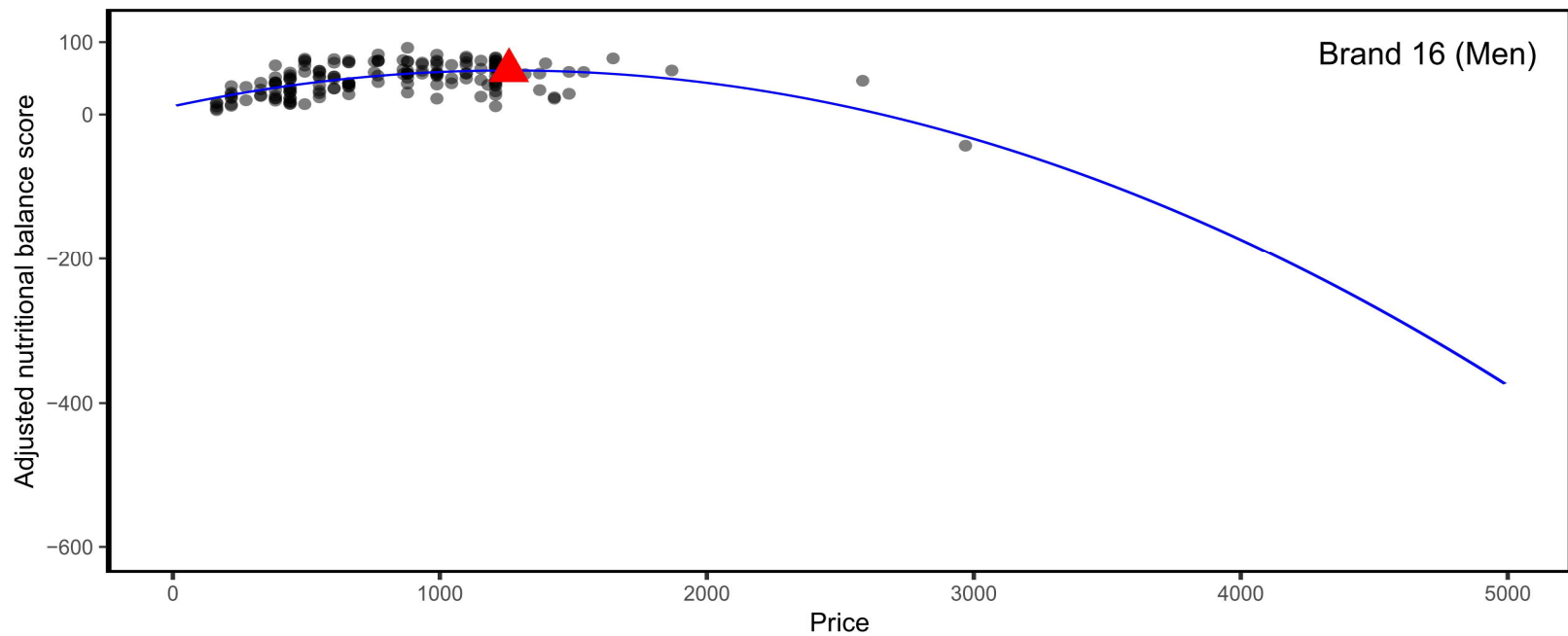

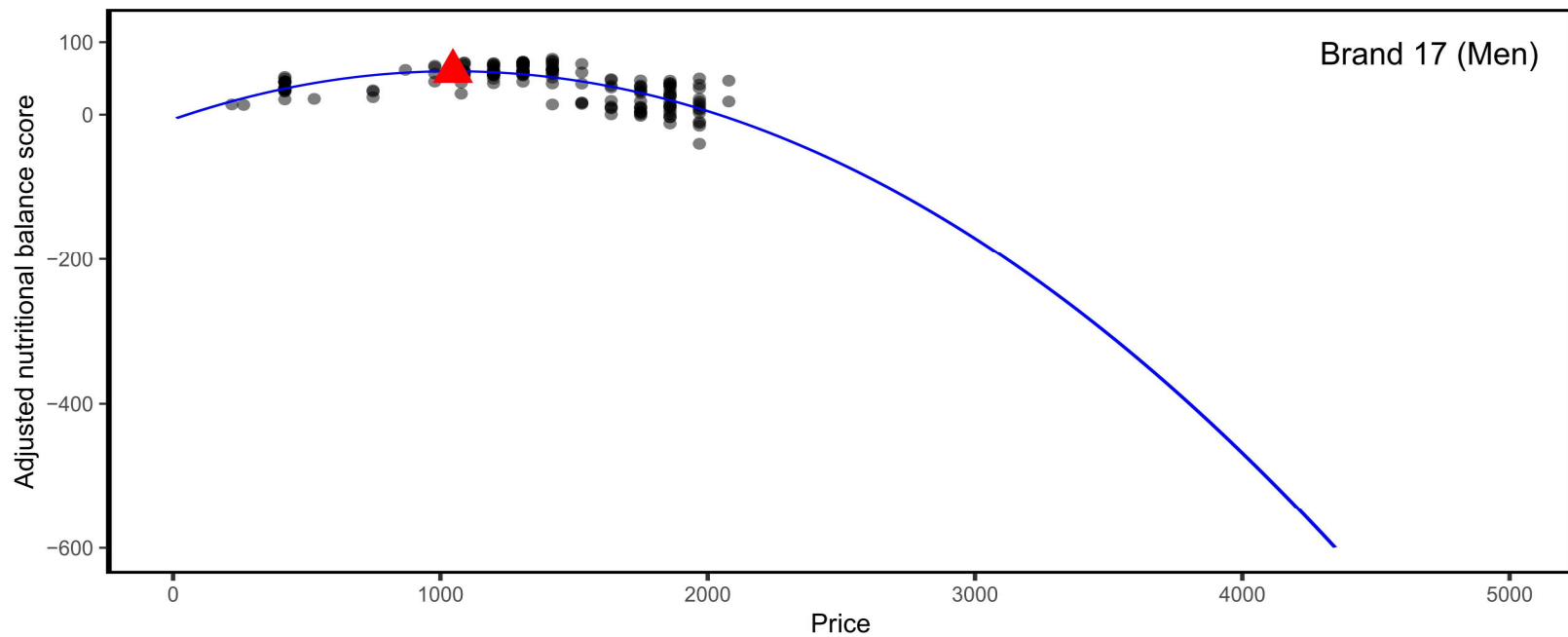

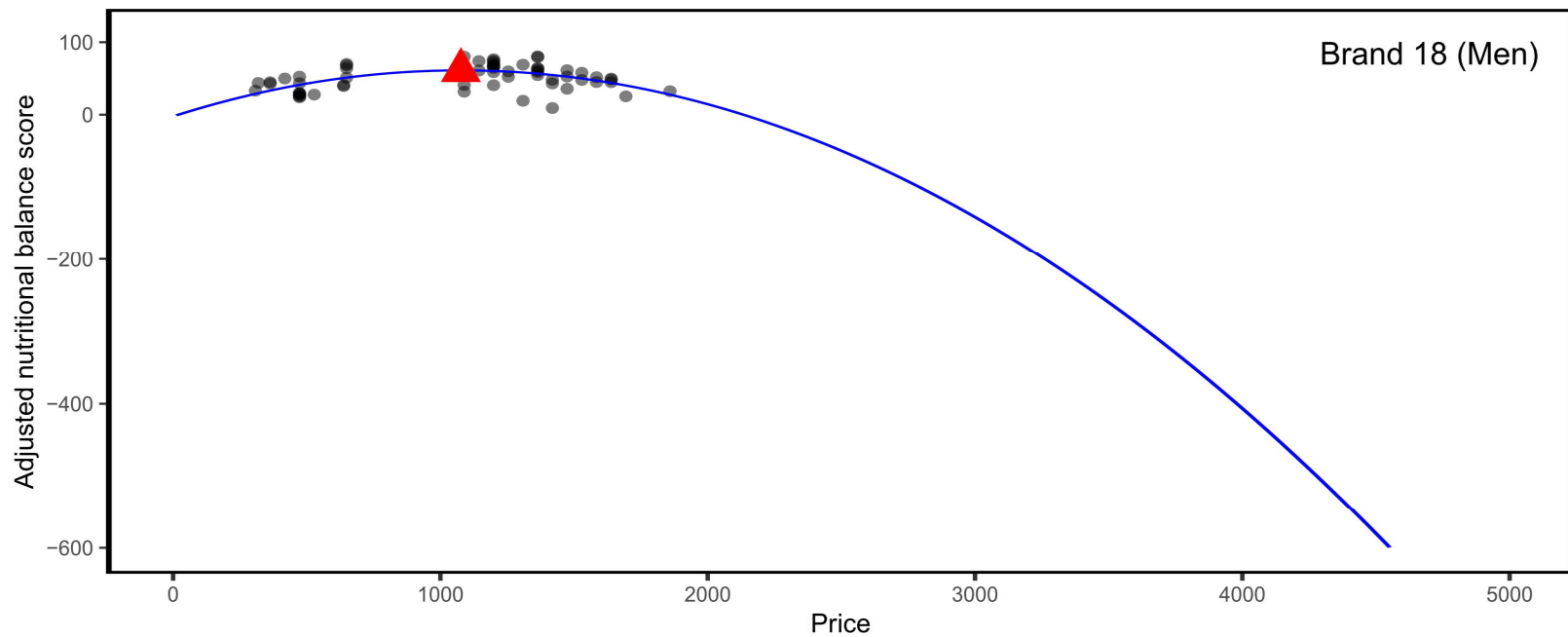

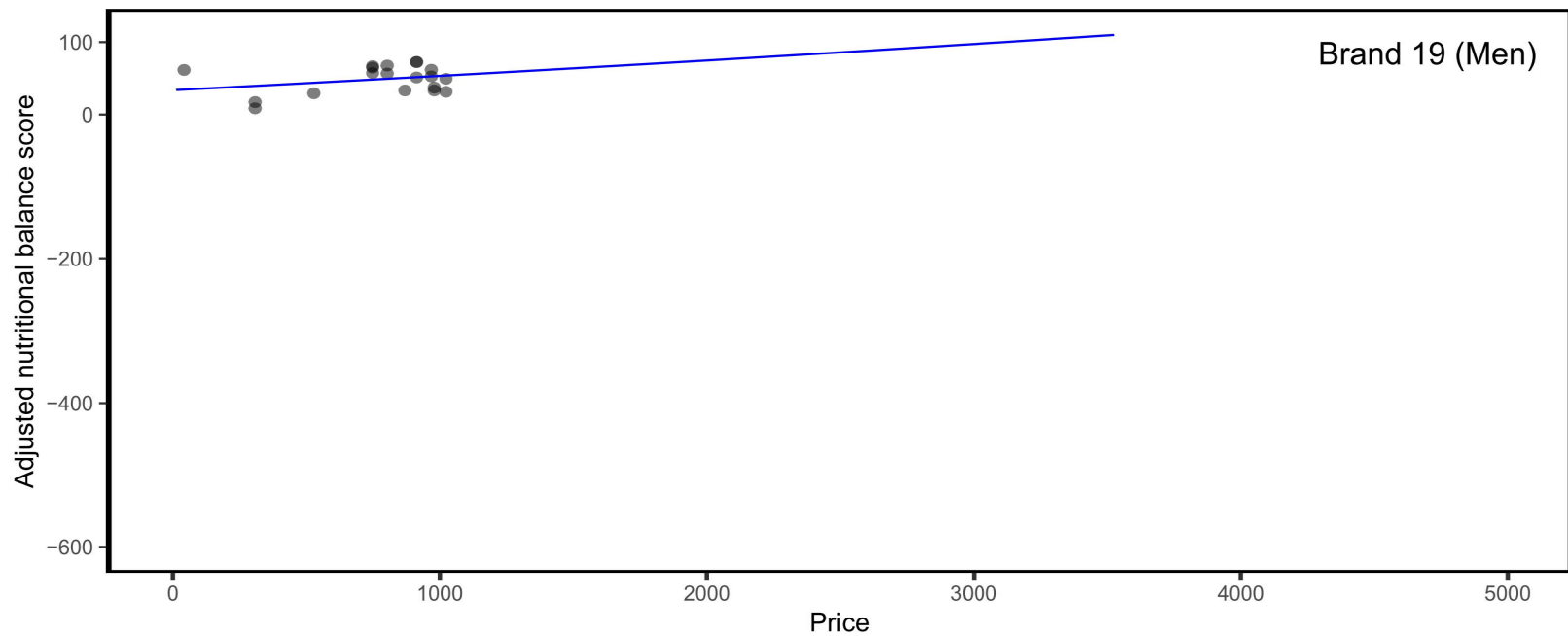

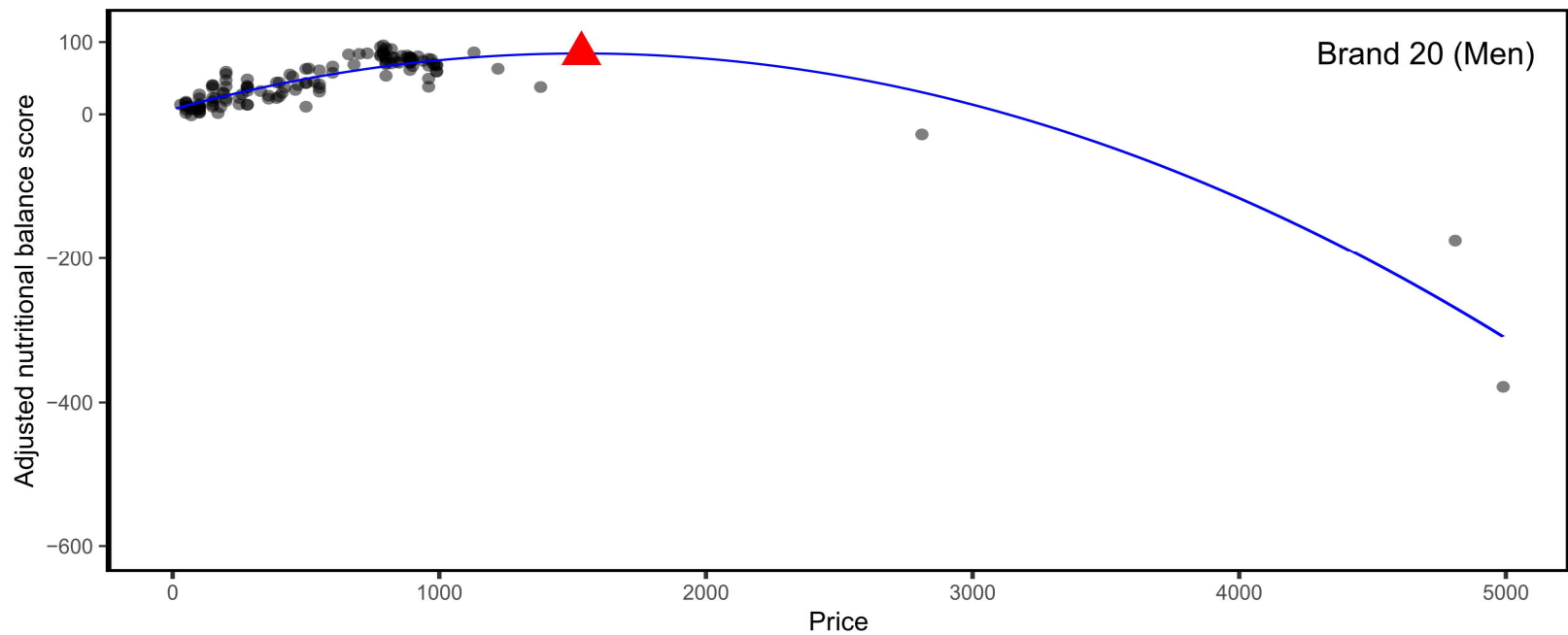

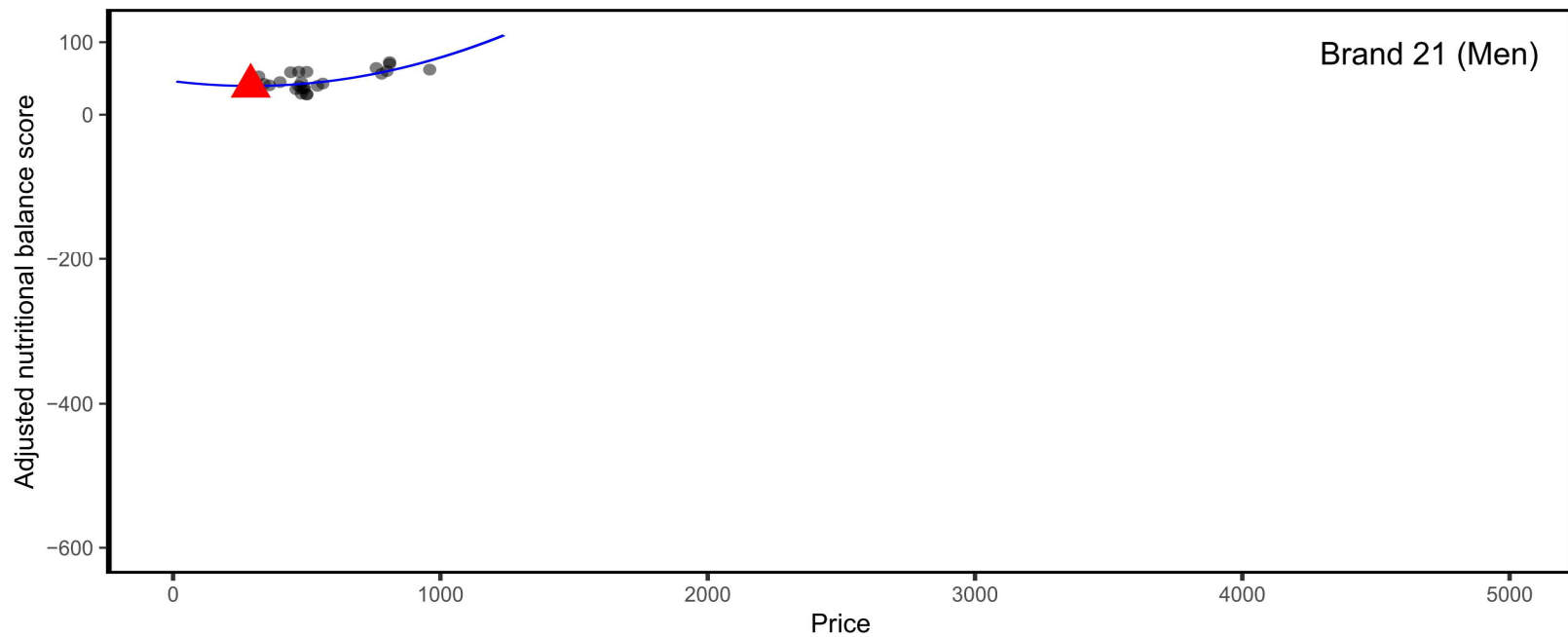

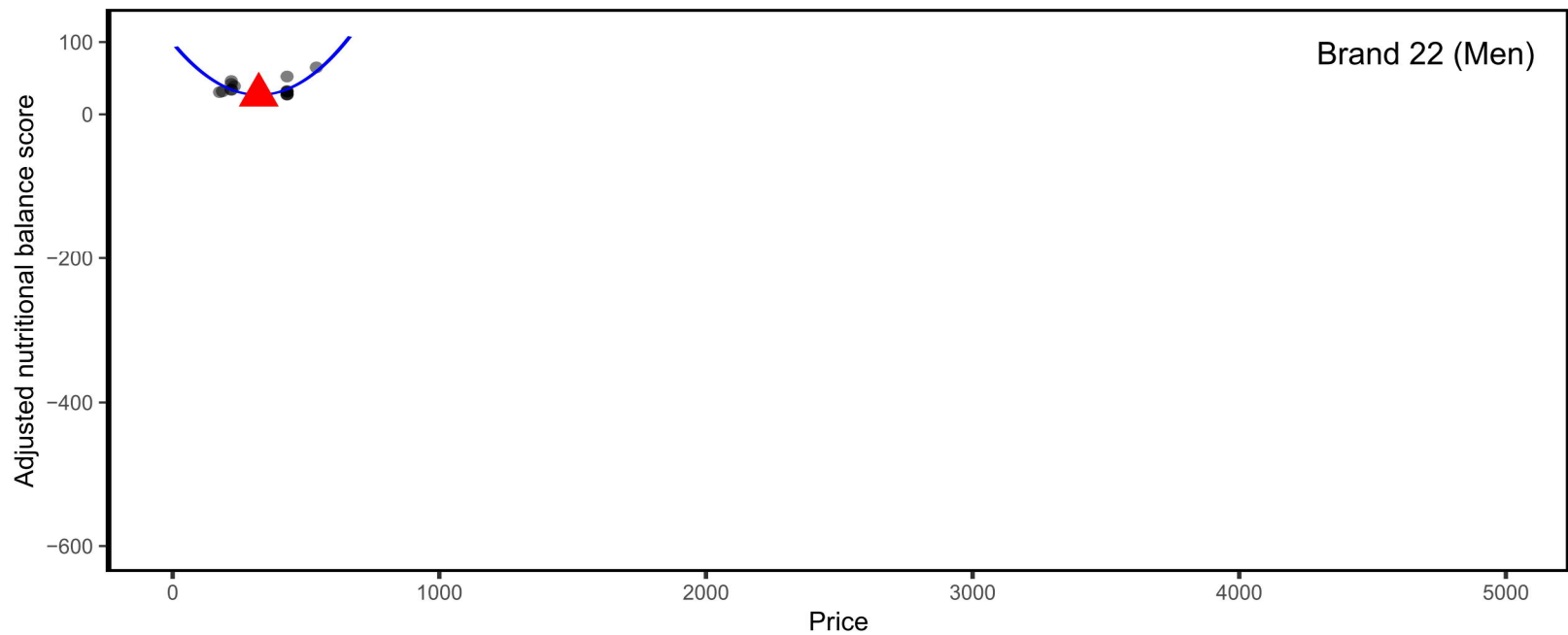

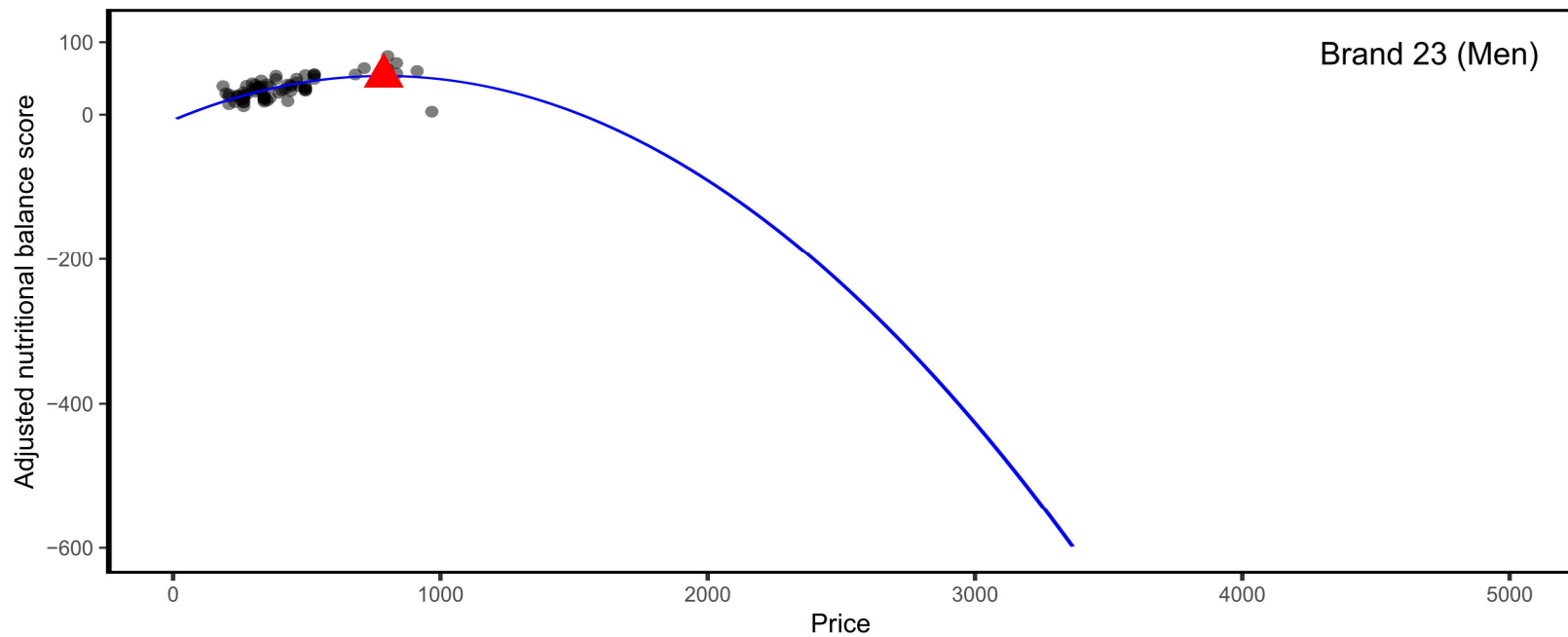

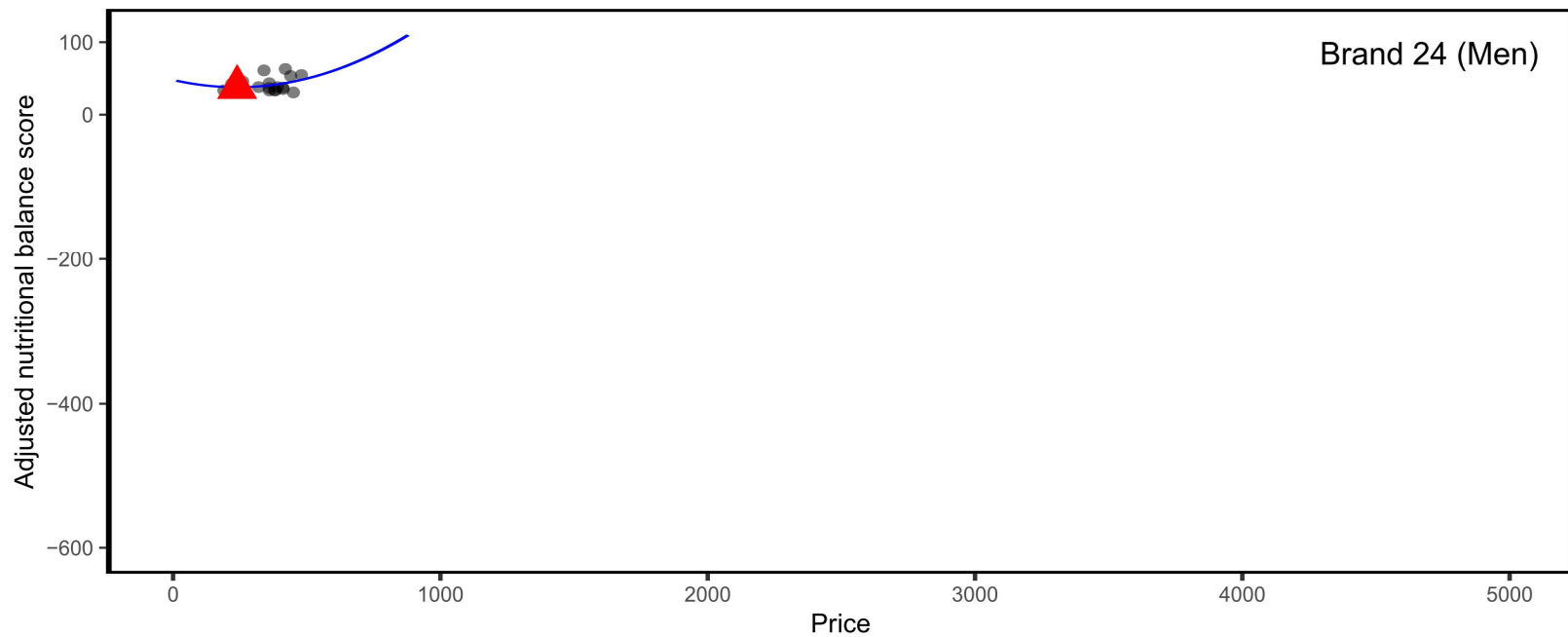

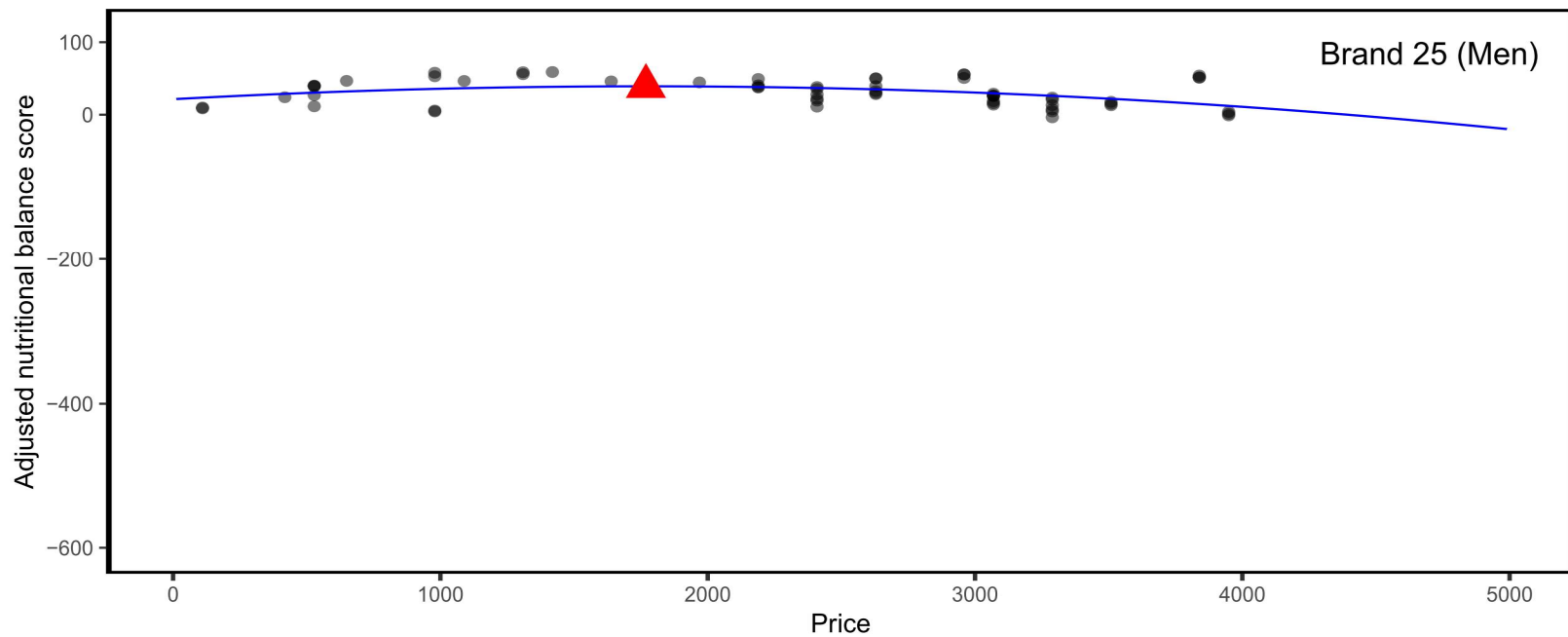

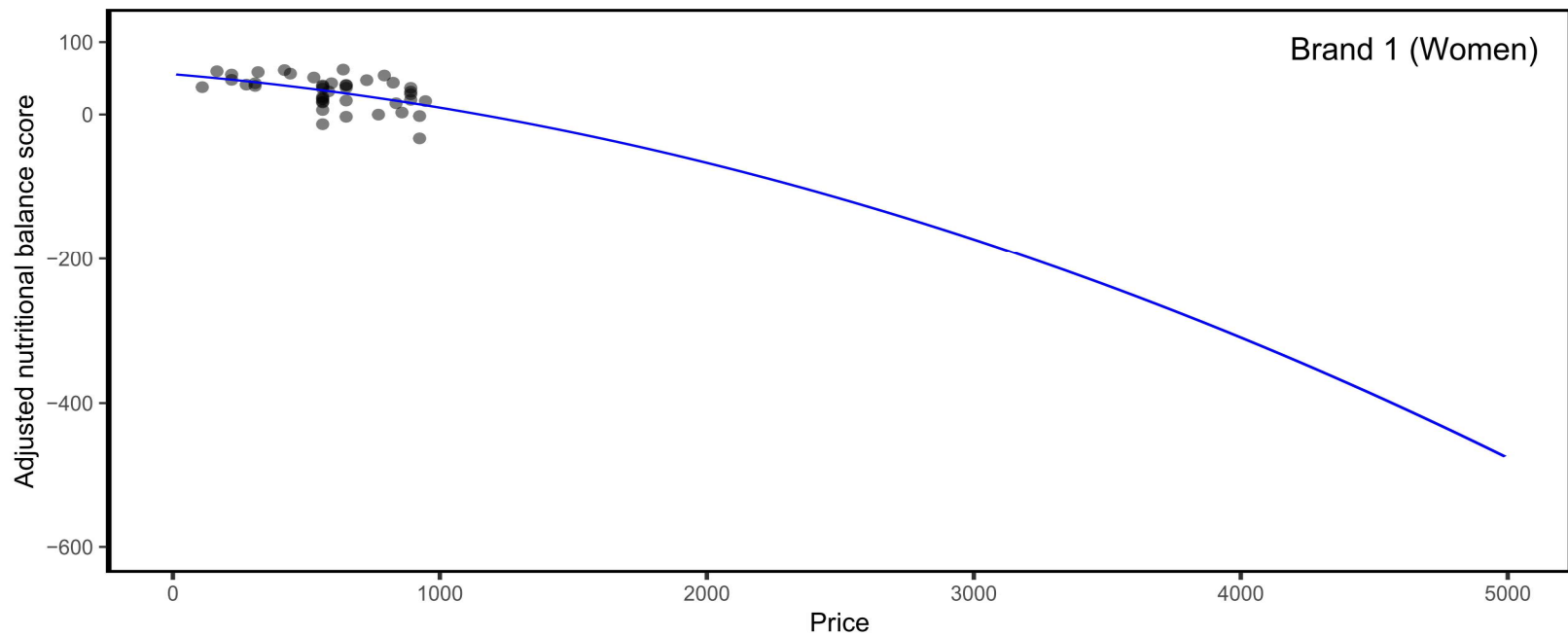

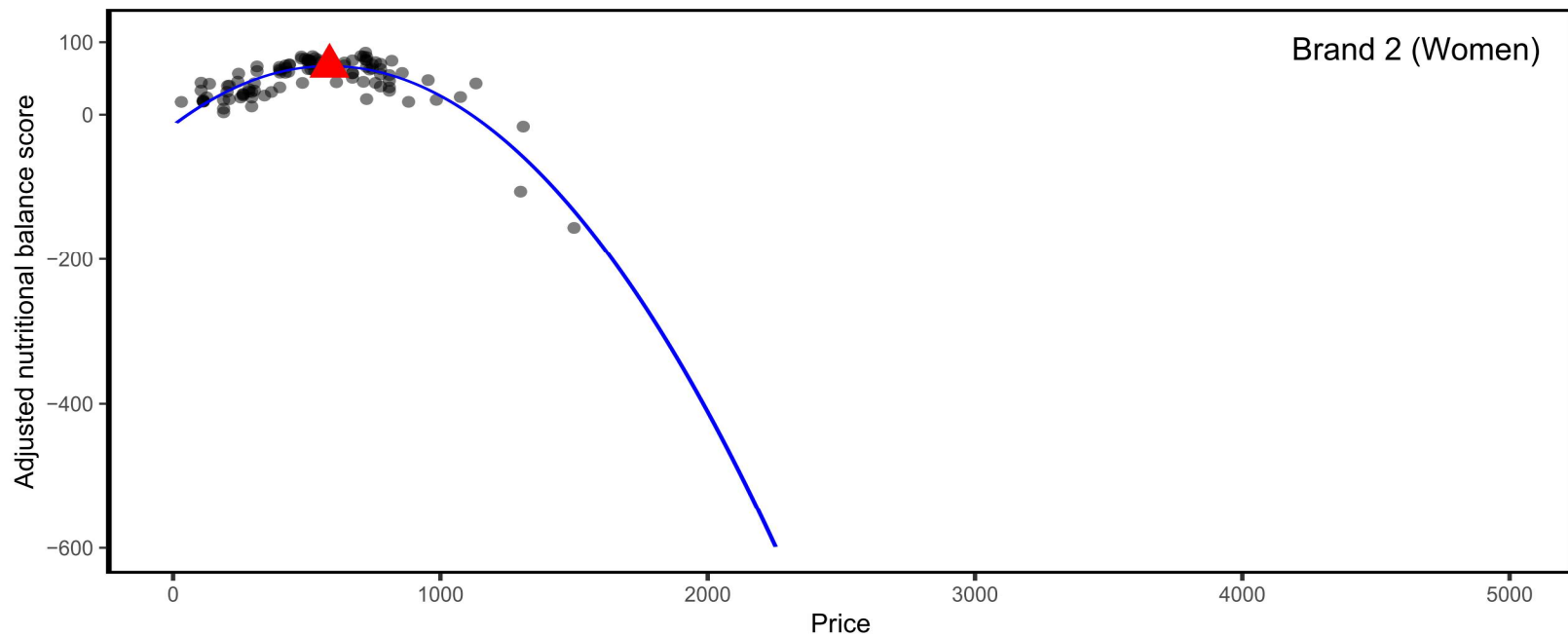

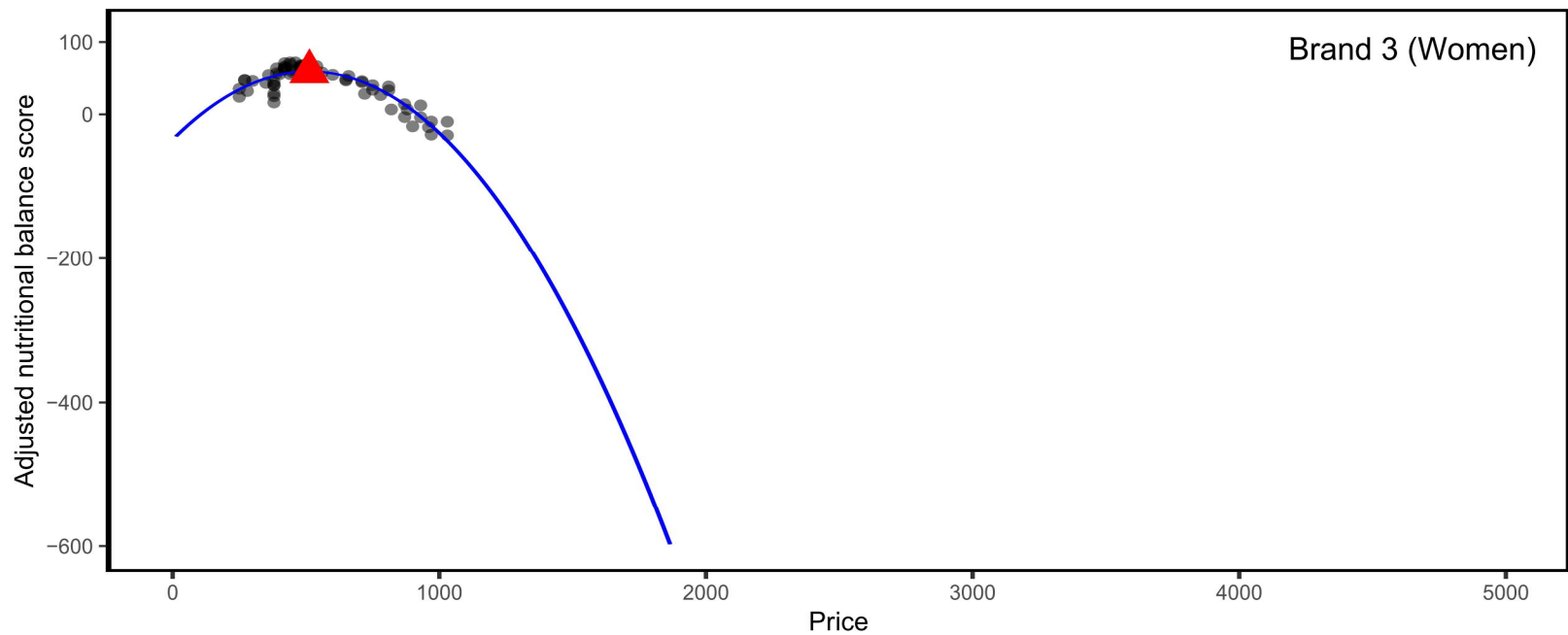

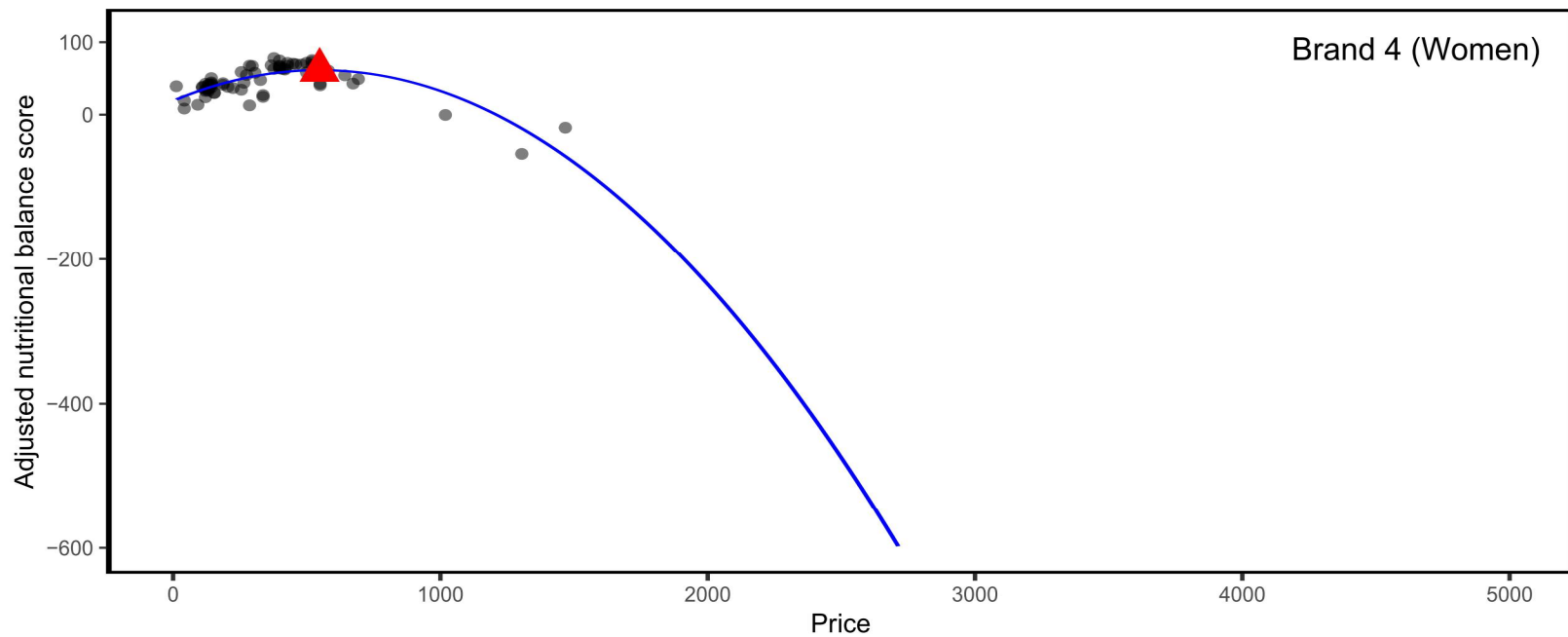

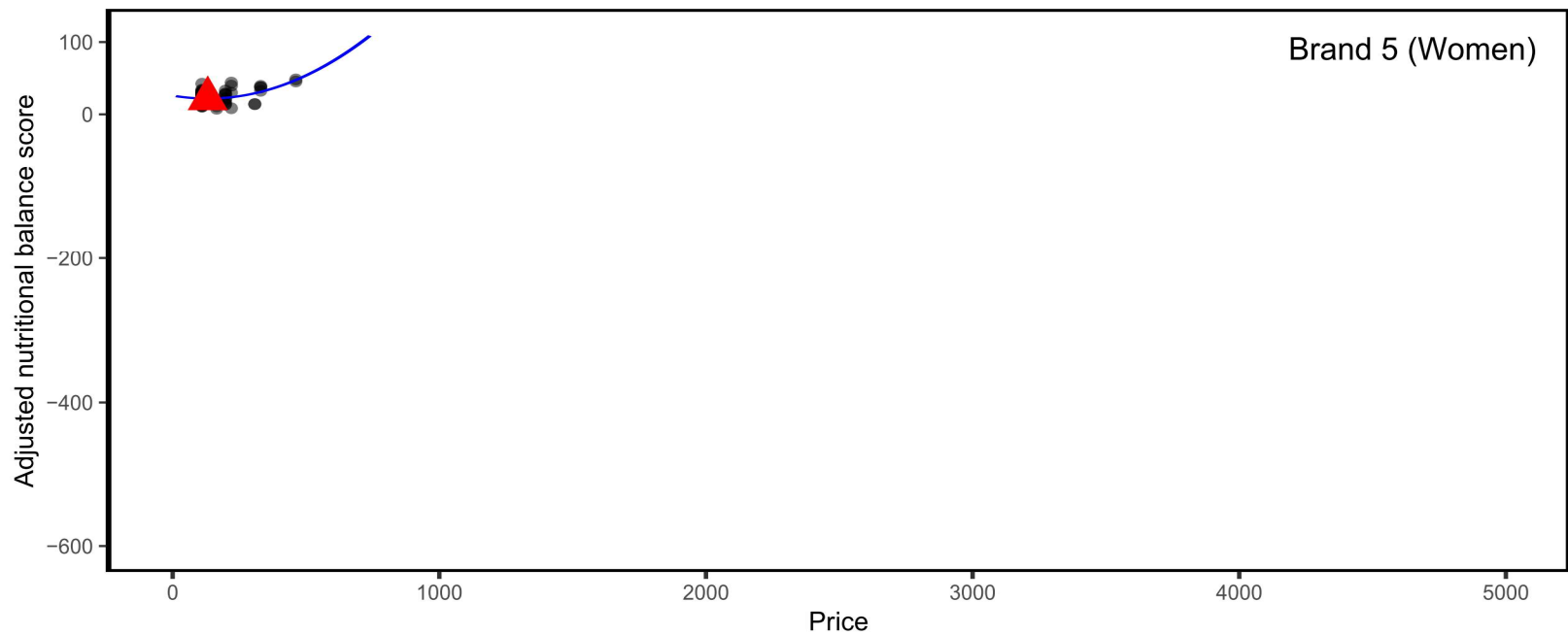

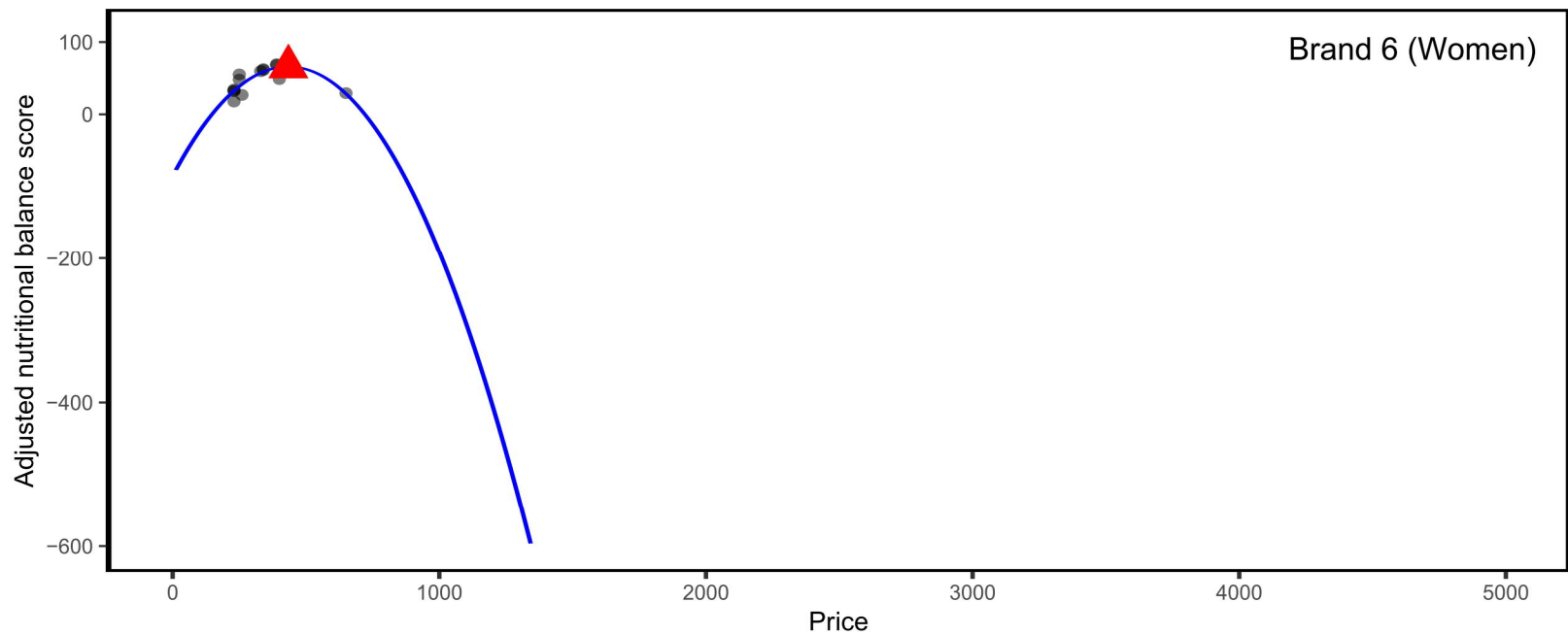

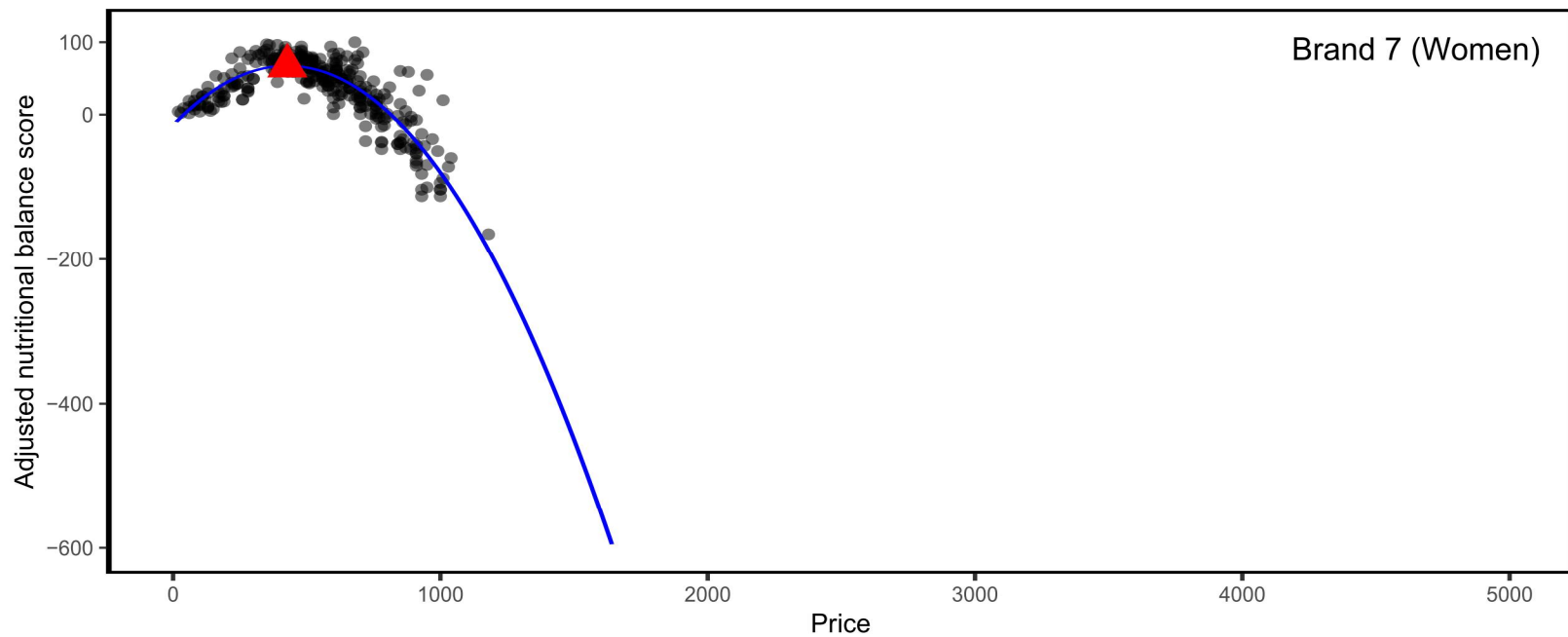

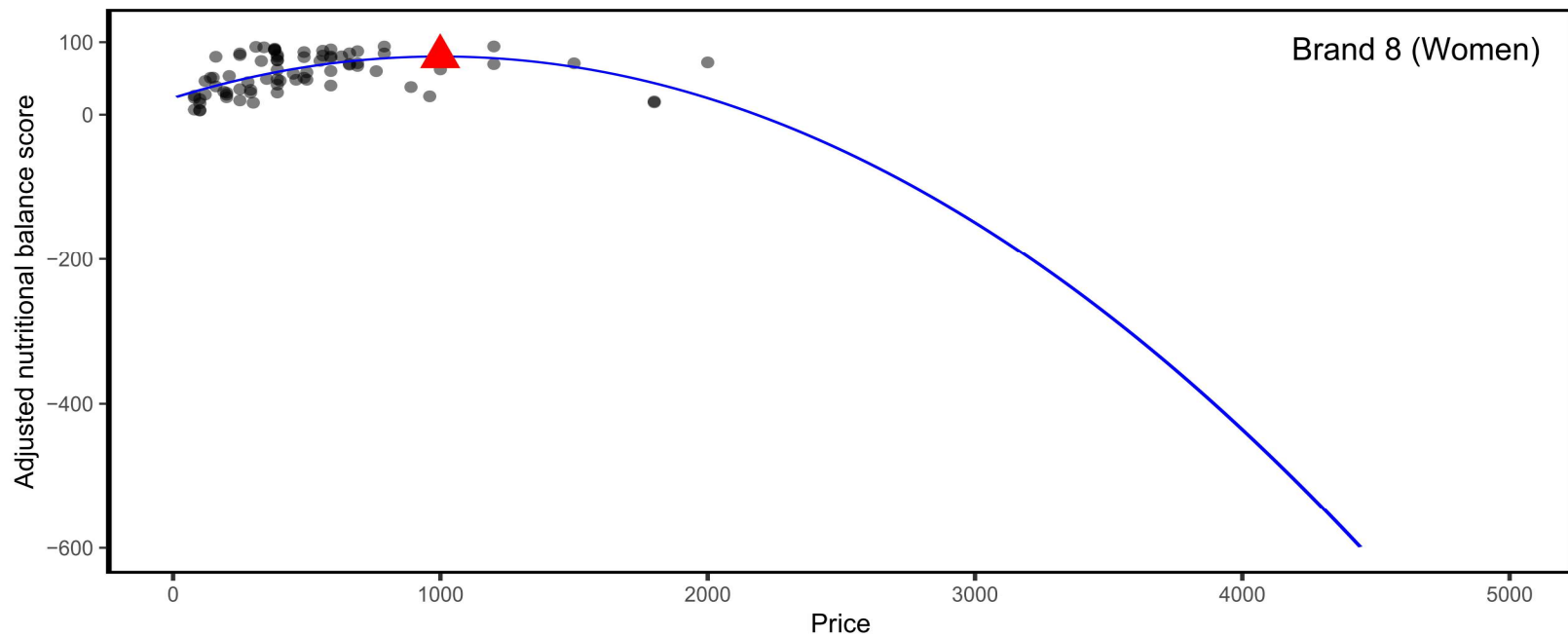

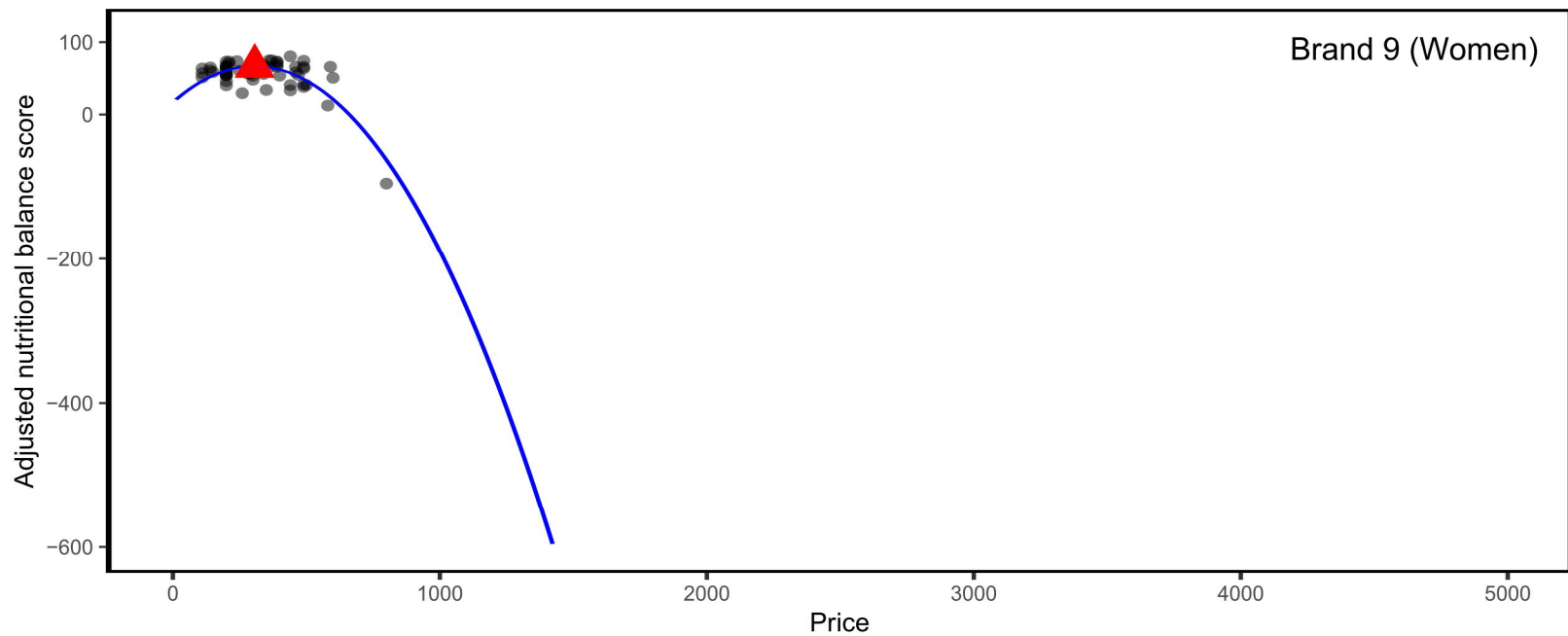

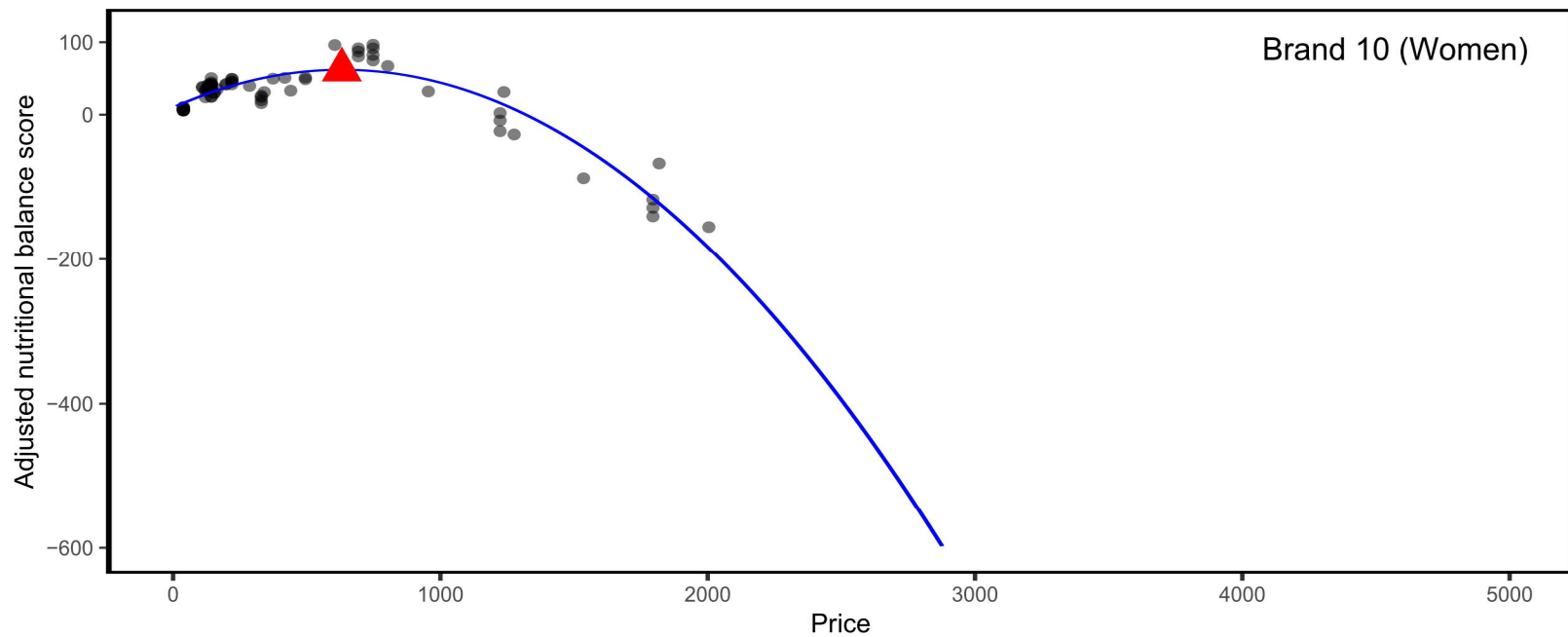

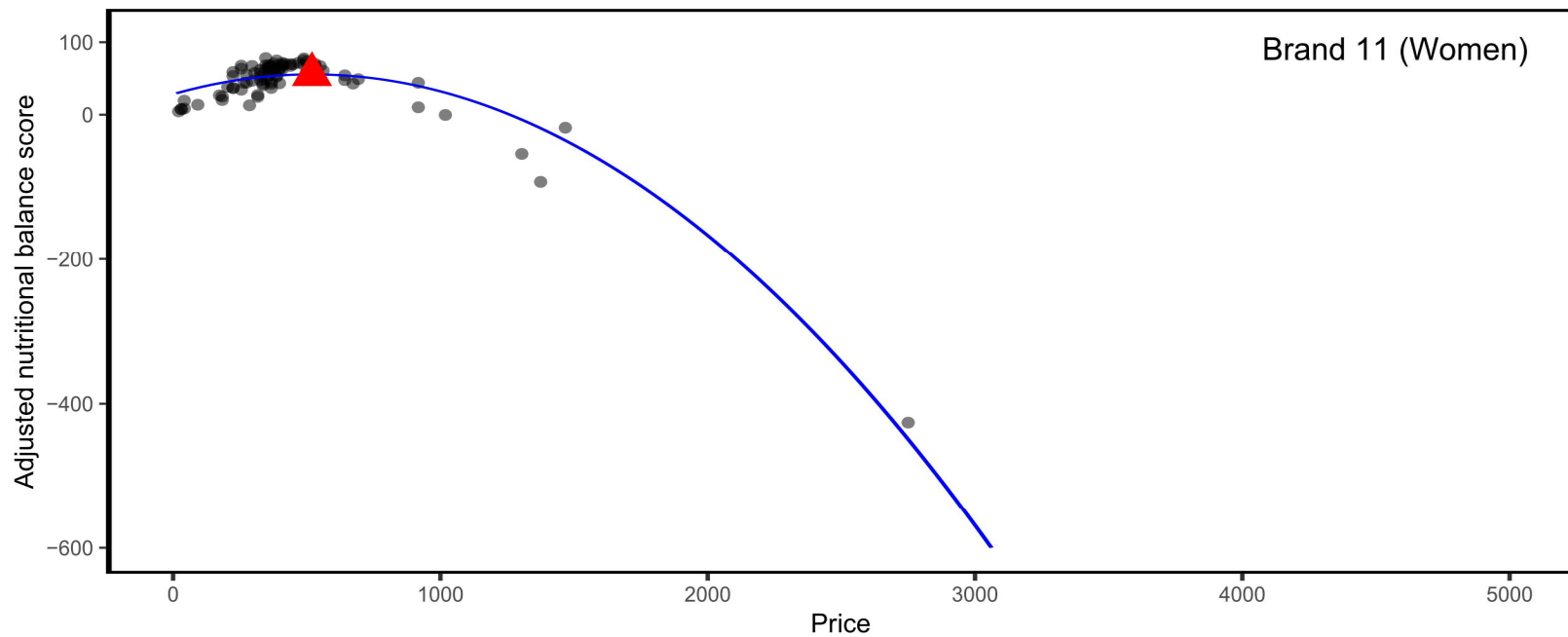

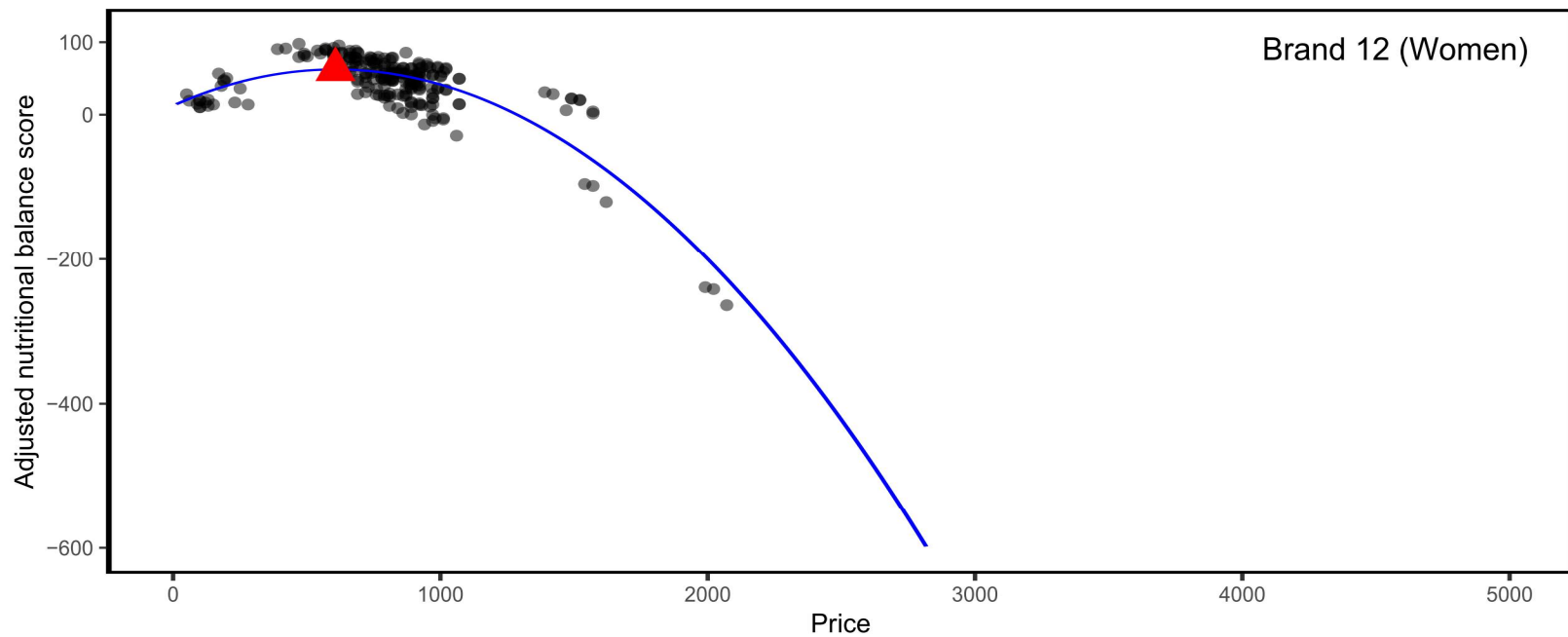

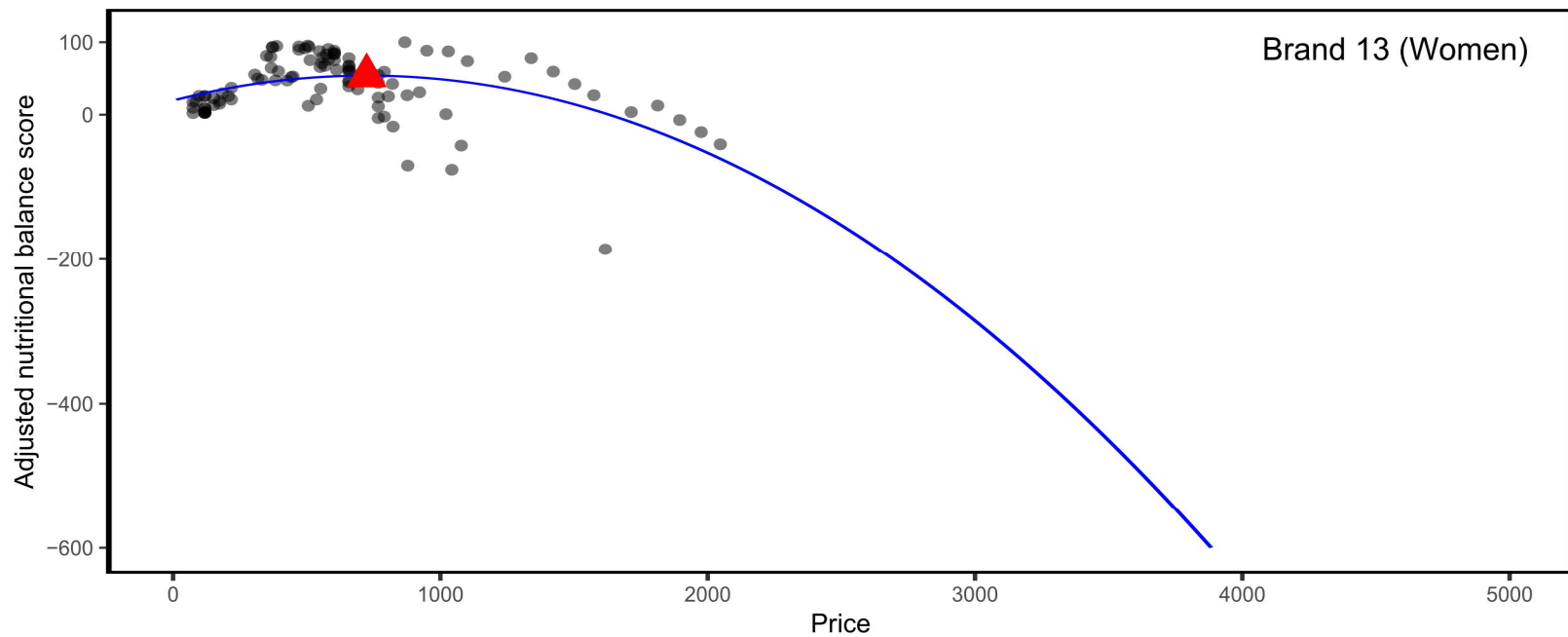

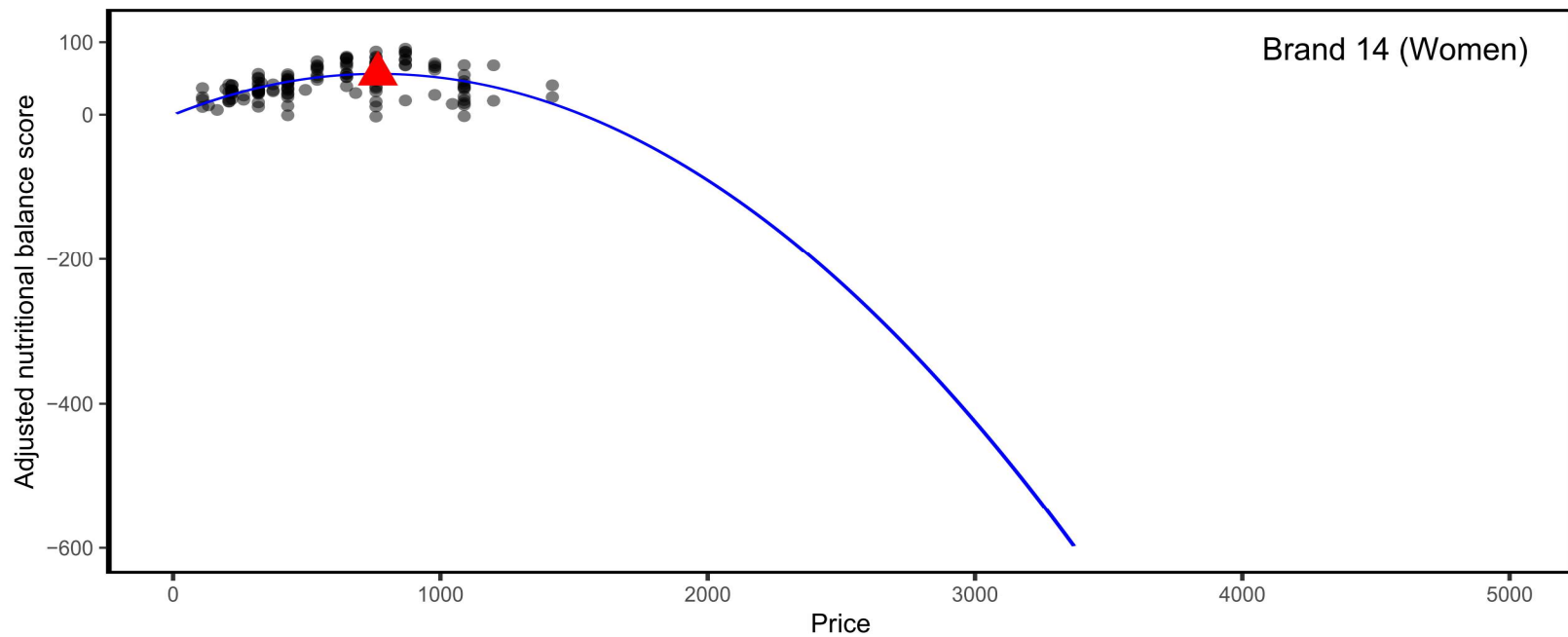

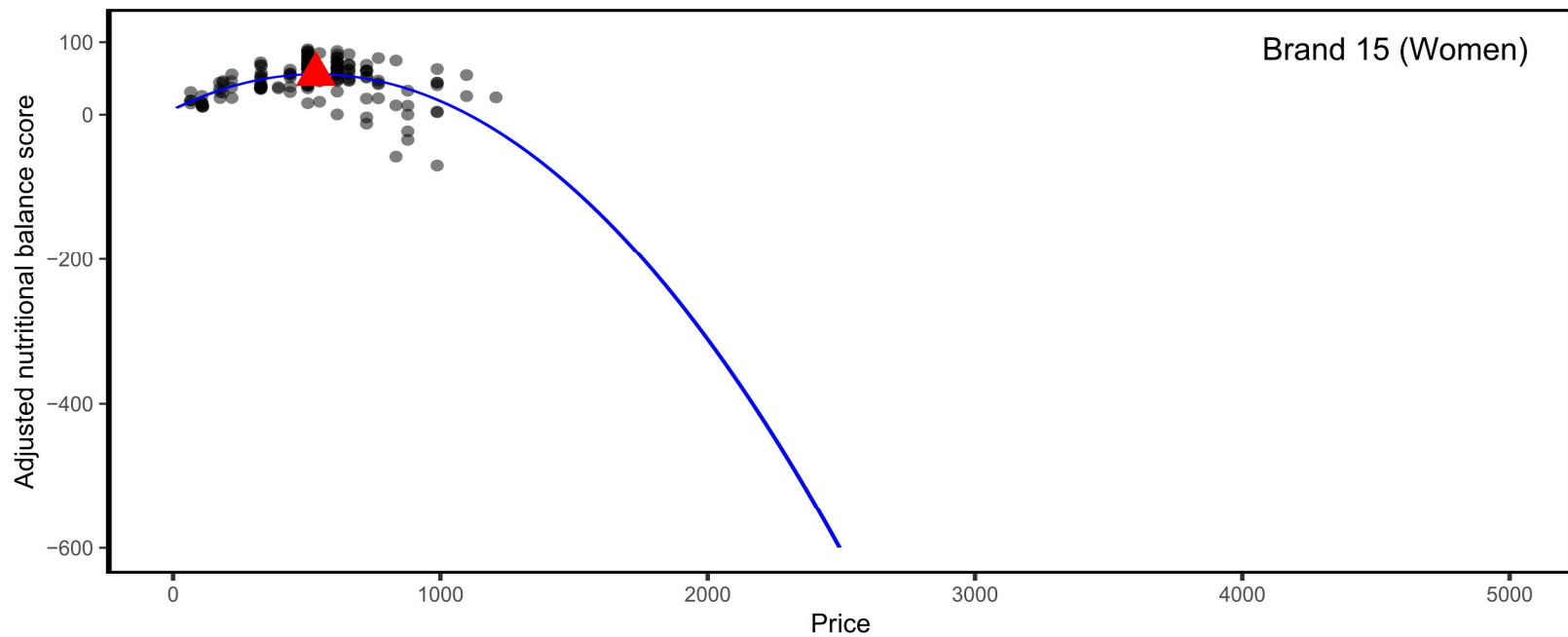

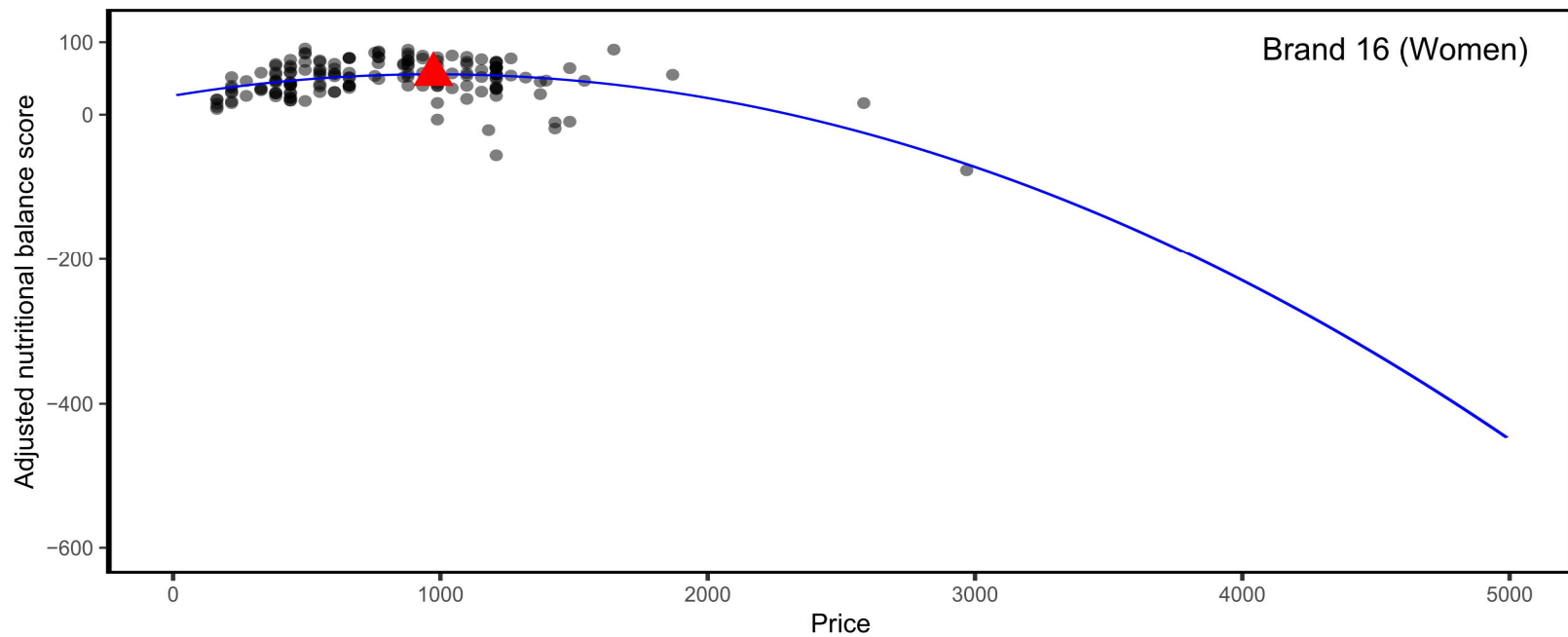

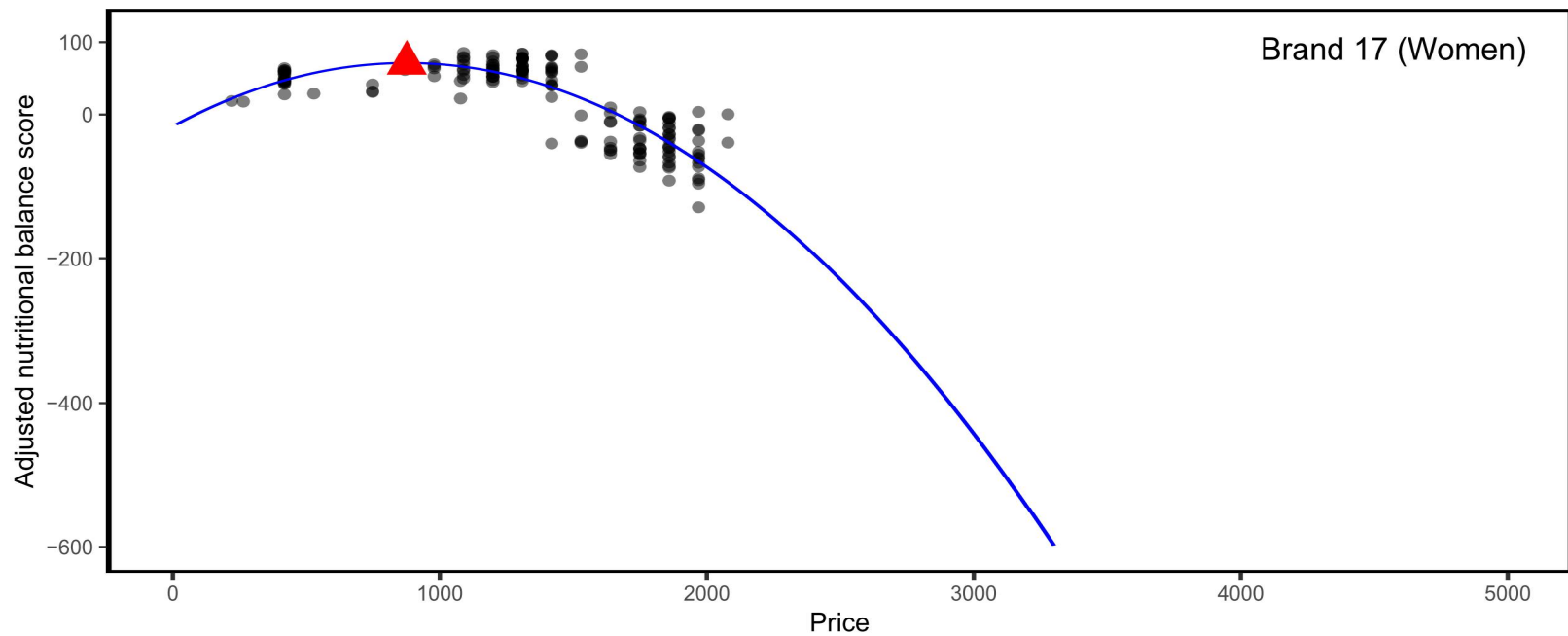

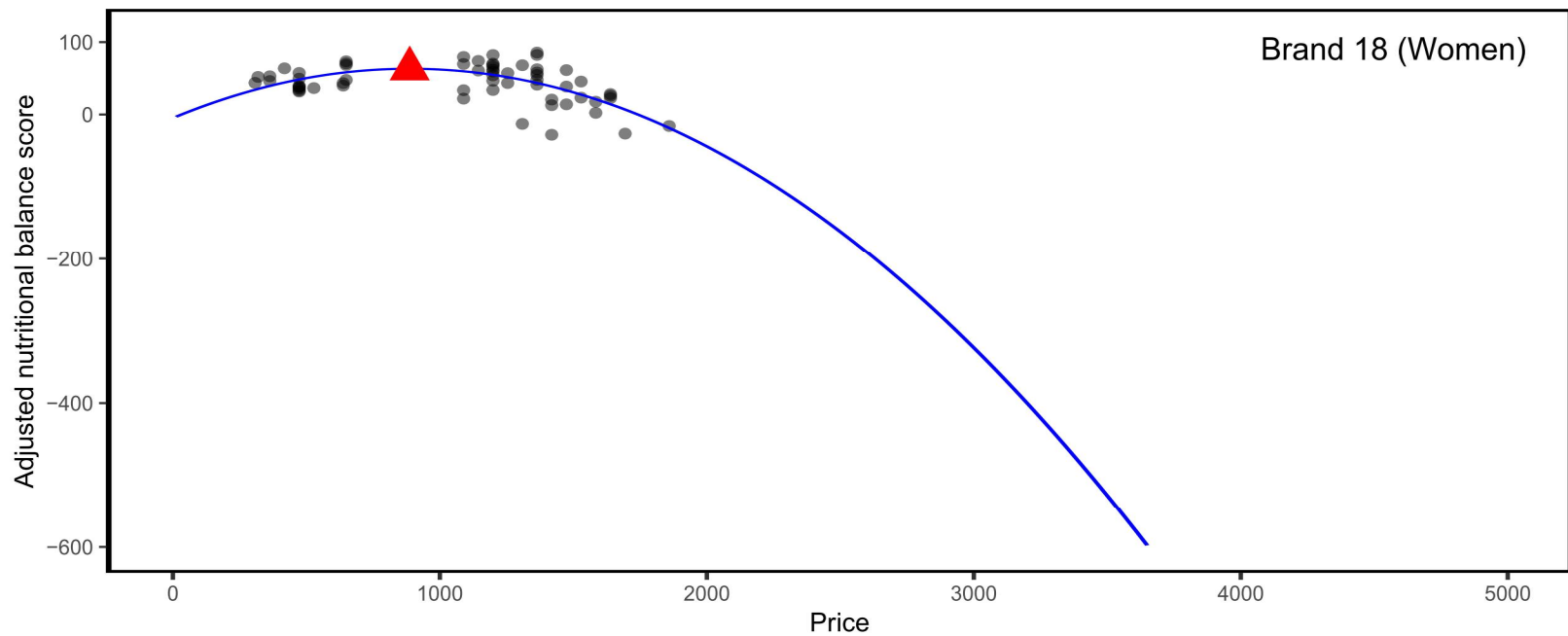

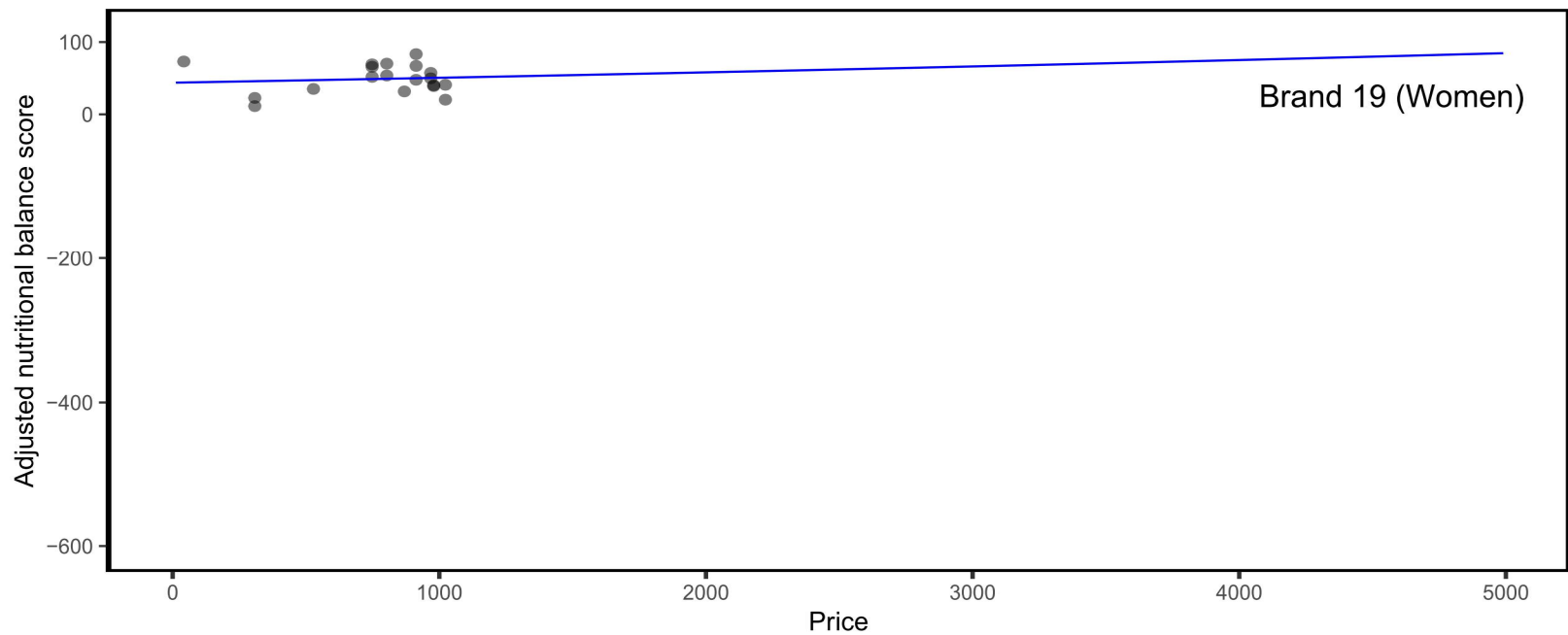

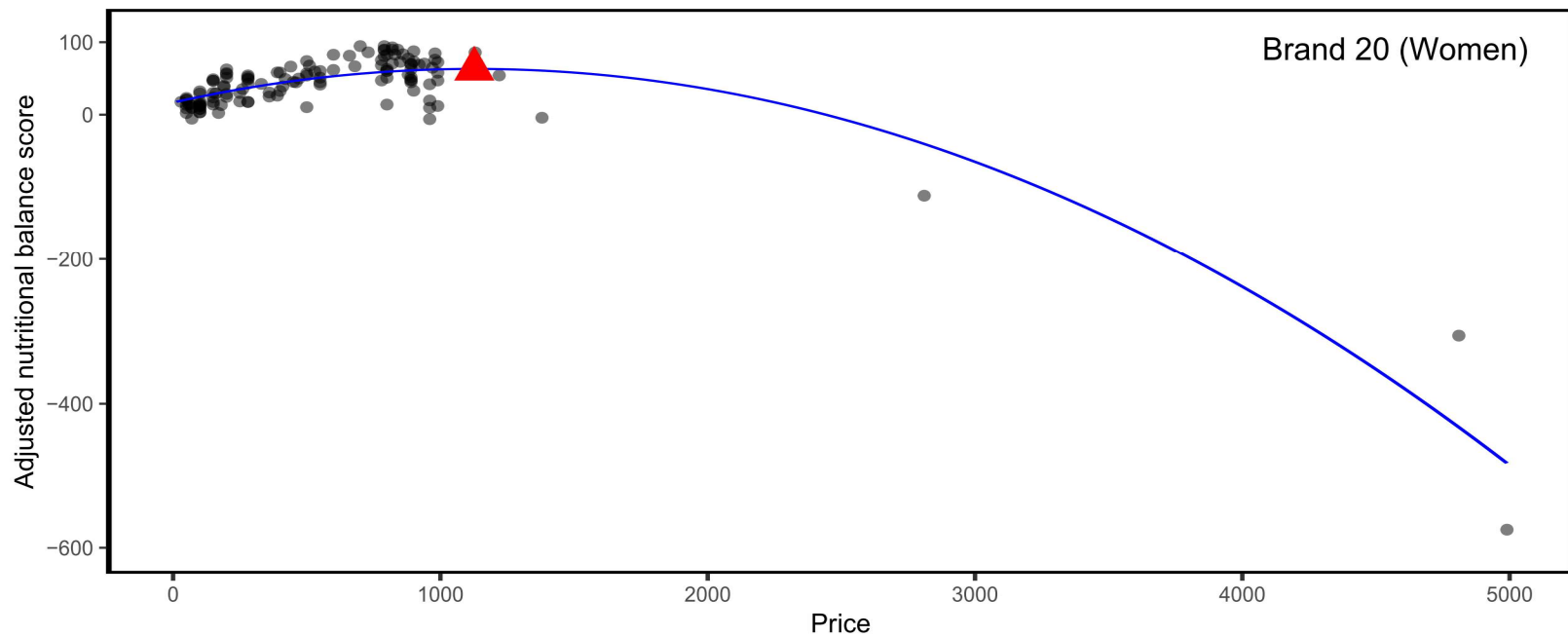

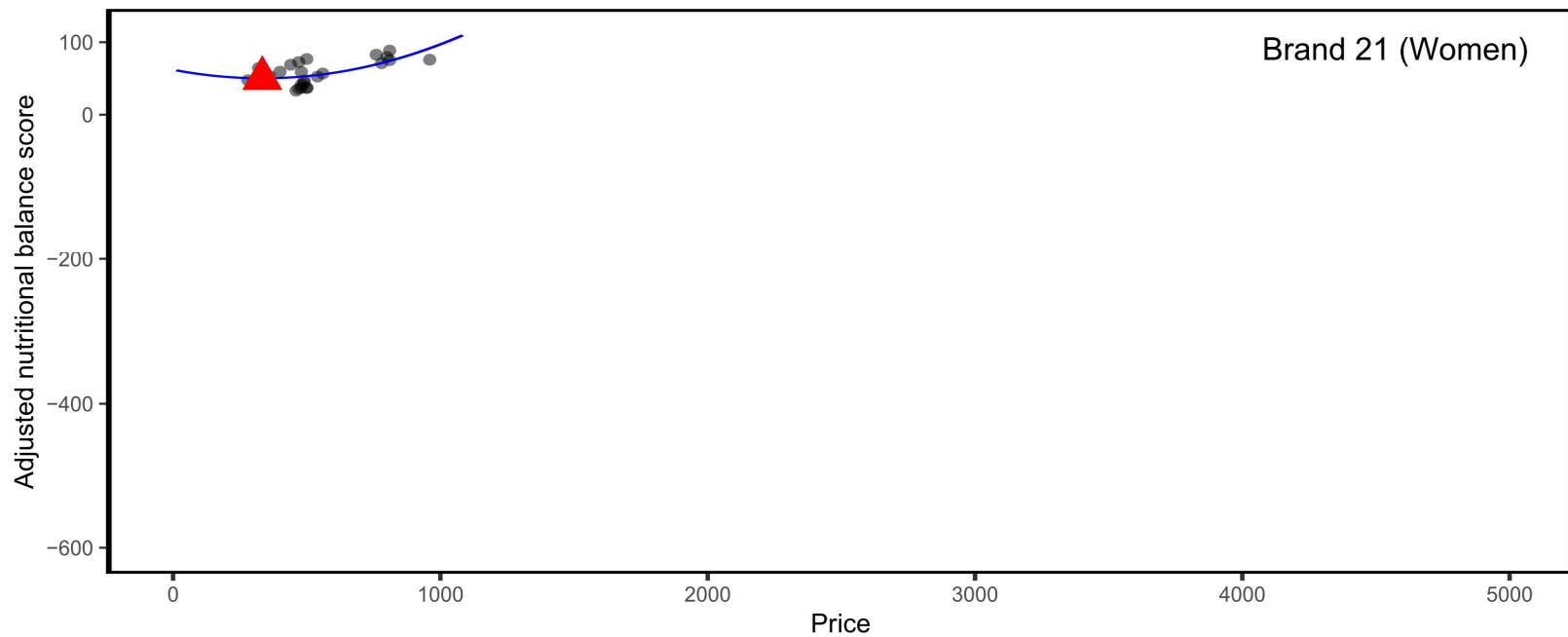

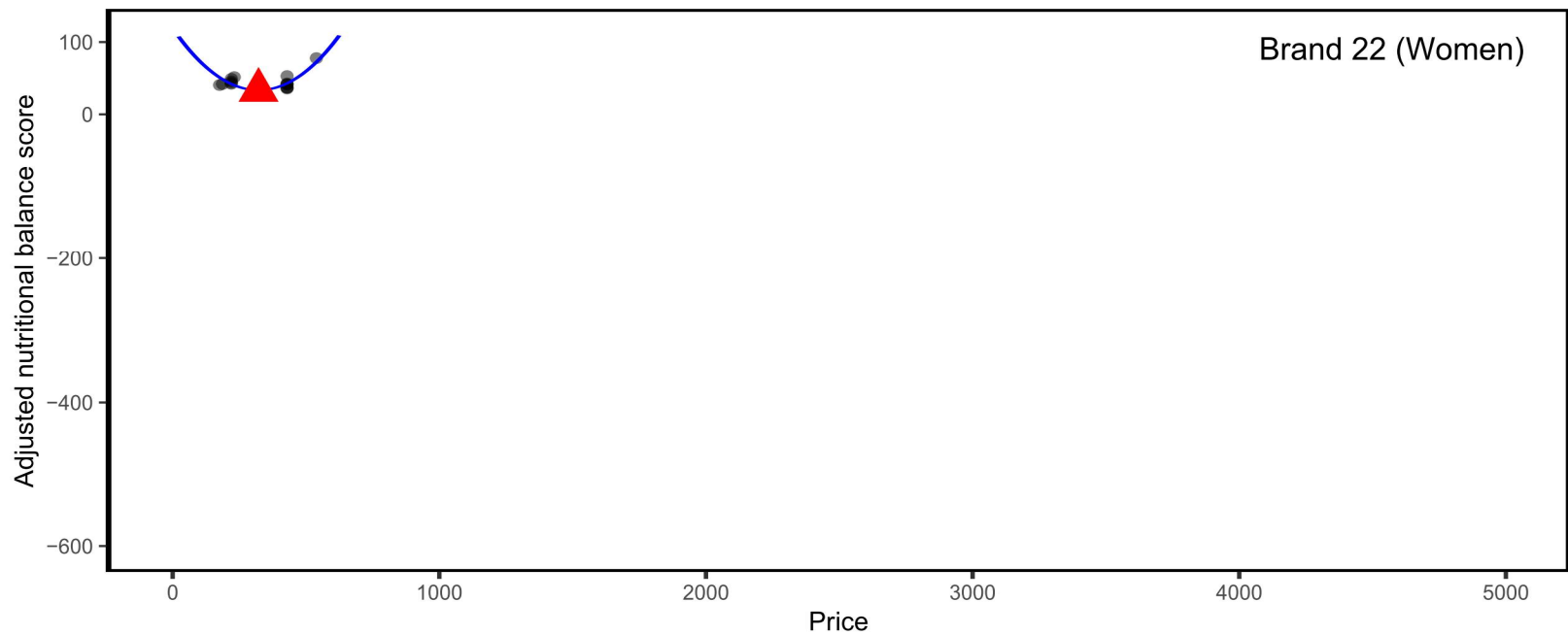

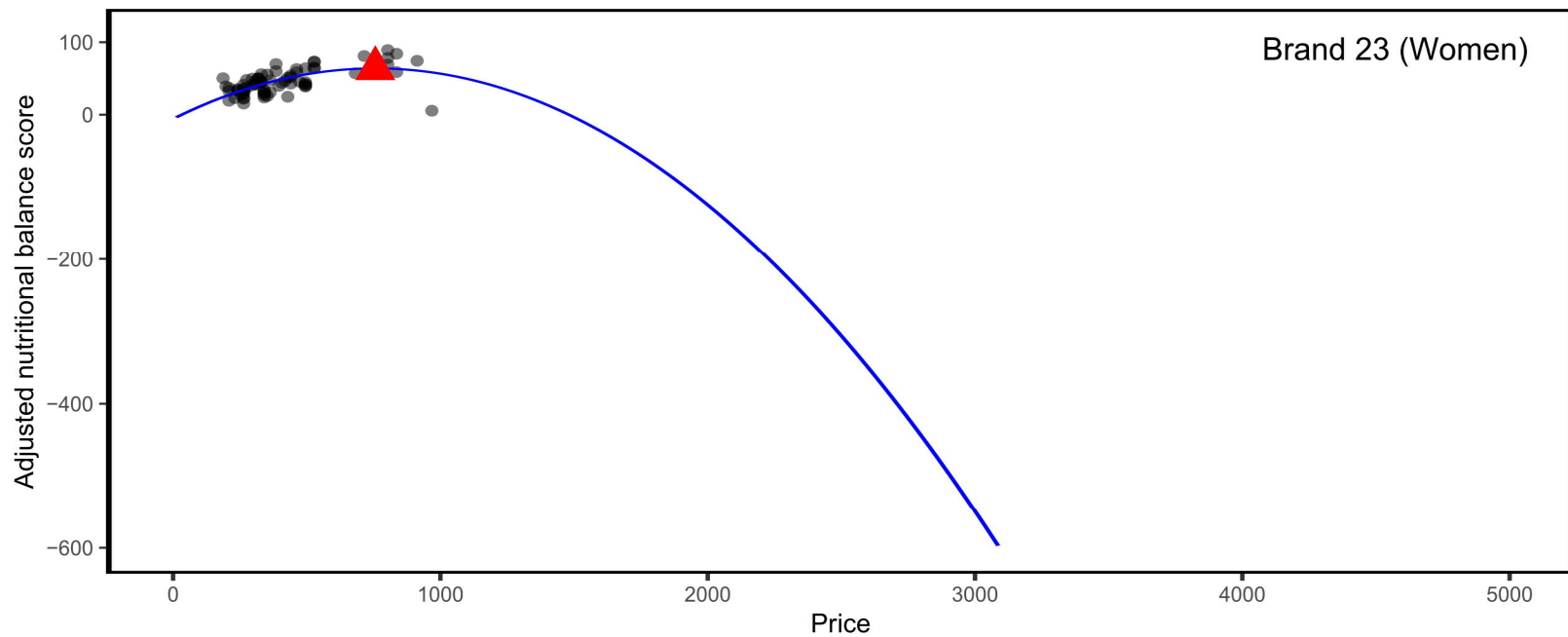

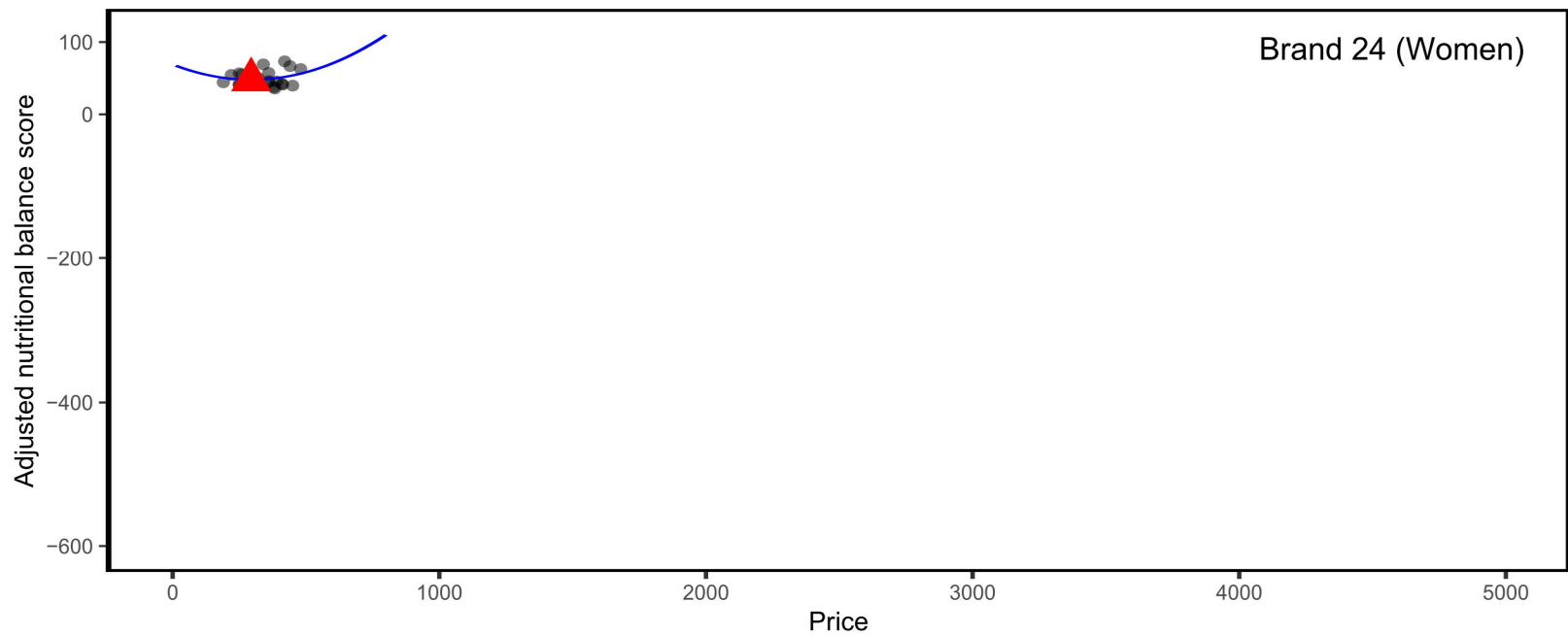

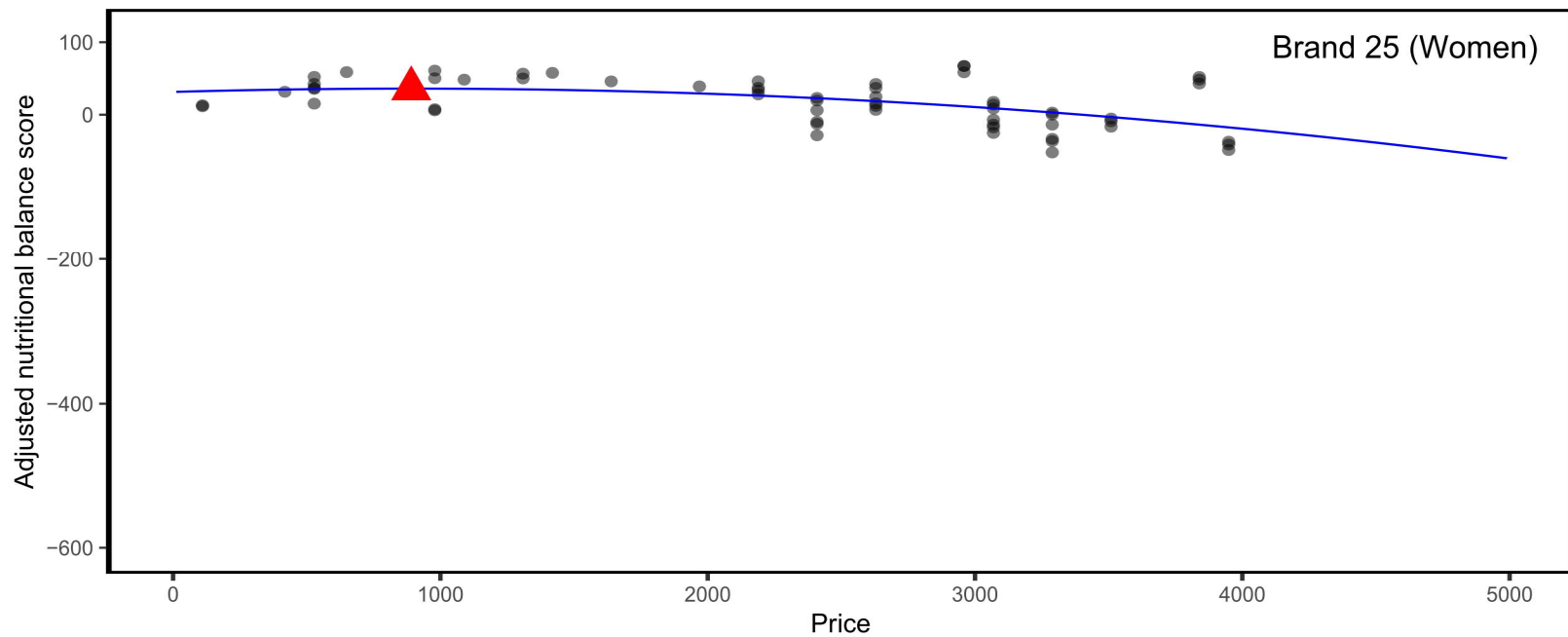

Supplement: Supplemental Information 2 [file peerj-12-18091-s002.pdf]
